# Physical exercise during neoadjuvant chemotherapy for breast cancer as a mean to increase pathological complete response rates: Trial protocol of the randomized Neo-ACT trial

**DOI:** 10.1371/journal.pone.0274804

**Published:** 2022-10-13

**Authors:** Jana de Boniface, Renske Altena, Cecilia Haddad Ringborg, Kate A. Bolam, Yvonne Wengström

**Affiliations:** 1 Department of Molecular Medicine and Surgery, Karolinska Institutet, Stockholm, Sweden; 2 Department of Surgery, Capio St. Göran’s Hospital, Stockholm, Sweden; 3 Department of Oncology-Pathology, Karolinska Institutet, Stockholm, Sweden; 4 Department of Oncology, Karolinska University Hospital, Stockholm, Sweden; 5 Department of Neurobiology, Care Sciences and Society, Division of Nursing, Karolinska Institutet, Stockholm, Sweden; 6 Karolinska Comprehensive Cancer Center, Karolinska University Hospital, Stockholm, Sweden; PhD, PLOS, UNITED KINGDOM

## Abstract

**Introduction:**

In early breast cancer, neoadjuvant chemotherapy (NACT) is increasingly used. The proof of efficacy is pathologically complete response (pCR), i.e. the absence of invasive tumour in breast and lymph nodes at surgery. Today, pCR is a common endpoint in pharmaceutical trials since it is significantly associated with survival especially in triple-negative and HER2-positive subtypes. Apart from the mitigation of treatment-related toxicity and symptoms, physical exercise mediates anti-tumoral systemic effects associated with tumour regression in preclinical and clinical models. The aim of Neo-ACT is to test the hypothesis that physical exercise can improve pCR rates in breast cancer patients receiving NACT.

**Method:**

The Neo-ACT trial is a prospective clinical trial, randomising T1-3N0-2 breast cancer patients planned for NACT to either a home-based physical exercise intervention supported by a mobile application or routine care. The primary endpoint is pCR; secondary endpoints are patient-reported quality of life, toxicity-related outcomes, and oncological outcomes such as Residual Cancer Burden, objective radiological tumour response, as well as overall, breast cancer-specific and disease-free survival at 2, 5 and 10 years. The intervention consists of a combination of high-intensity interval and resistance training of progressing intensity, and includes at least 150 min of moderate to vigorous physical activity per week, inclusive of two weekly 60-min exercise sessions. In order to show an improvement in pCR of 10%, a total of 712 participants need to be included in the analysis. The Neo-ACT has been registered at clinicaltrials.gov on January 11, 2022 (NCT05184582).

**Expected results:**

If Neo-ACT can prove the oncological efficacy of physical exercise, implementation of training programmes into NACT schedules will be pursued. The use of a digitally led exercise intervention aims to test the potential of such a strategy for use in rural areas and areas of limited resources.

## Introduction

Neoadjuvant chemotherapy (NACT) is increasingly used for patients with early breast cancer (BC) in accordance with international guidelines [1]. The best proof of NACT efficacy is pathological complete response (pCR), i.e. the absence of residual invasive tumour in the breast and the axillary lymph nodes (ypT_0/is_N_0_). In triple-negative (TNBC) and HER2-positive BC, published pCR rates are highest and strongly predict improved survival, while pCR rates are substantially lower in luminal BC (ER-positive, HER2-negative) and have a less obvious prognostic value [2]. Today, pCR is frequently used as surrogate endpoint in oncological pharmaceutical trials focusing on new compounds.

There is epidemiological evidence for energy intake as a risk factors for breast cancer, counterbalanced by physical activity [3]. In BRCA1 and 2 carriers, physical activity and lack of obesity in adolescence is associated to later onset of cancer, suggesting a protective effect [4]. Preclinical studies have shown that physical exercise reduces tumour growth in animal models of breast cancer [5]. Exercise acts through reduced systemic inflammation (reflected by lower C-reactive protein and pro-inflammatory cytokine levels in serum), enhanced anti-tumoural immune cell function and increased recruitment and cytotoxic activity of CD8+ T-cells and NK cells, a shift towards an anti-tumorigenic (Th1/M1) profile and an altered phenotype of tumour vasculature, improving blood flow and perfusion, and making the tumour more susceptible to systemic treatment [6–9]. In the tumour microenvironment, the level of inflammatory cell infiltration increases markedly in response to physical exercise [10]. Primary tumour growth is reduced in mice exposed to voluntary running [11, 12]. Recently, a non-randomized prospective trial evaluated physical exercise during neoadjuvant treatment in patients with oesophageal cancer; participants in the exercise group had significantly more pronounced tumour regression at surgery than those in the control group [13].

The safety and feasibility of physical exercise during chemotherapy has been demonstrated both under supervision and when performed via tailored home-based exercise sessions during neoadjuvant chemotherapy [13–15]. Evidence from observational studies shows that exercise following a BC diagnosis has a protective effect on recurrence as well as all-cause and cancer-specific mortality [16]. Furthermore, long-term follow-up of a randomized exercise intervention consisting of aerobic and resistance exercise showed enhanced disease-free and overall survival in patients with breast cancer [17, 18]. In the randomized OptiTrain trial, resistance and high-intensity interval training (HIIT) during postoperative chemotherapy had positive effects on cancer related fatigue, cardiorespiratory fitness, muscle strength [19], and muscle mass and function [20]. Chemotherapy completion rates, which are strongly associated with an improved prognosis [21], can be improved by an exercise program of combined resistance and aerobic training [22]. Physical exercise may thus result in improved pCR rates after NACT not only through proposed systemic anti-inflammatory effects, but also through improved chemotherapy completion rates given at full dosage due to the favourable effects of exercise on fatigue, muscle strength and cardiorespiratory fitness which drive improvements in treatment tolerability. Thus, there is great potential for physical exercise to be put forward as a feasible and effective strategy to support patients to tolerate treatments, which needs to be corroborated in prospective trials. The effect of physical exercise during NACT on pCR rates in breast cancer has however never been tested. The Neo-ACT trial is a multicentre phase 3 randomized controlled trial that aims to explore the efficacy of physical exercise for improvement of oncological outcomes on a clinically relevant scale.

## Materials and methods

**Fig 1:**
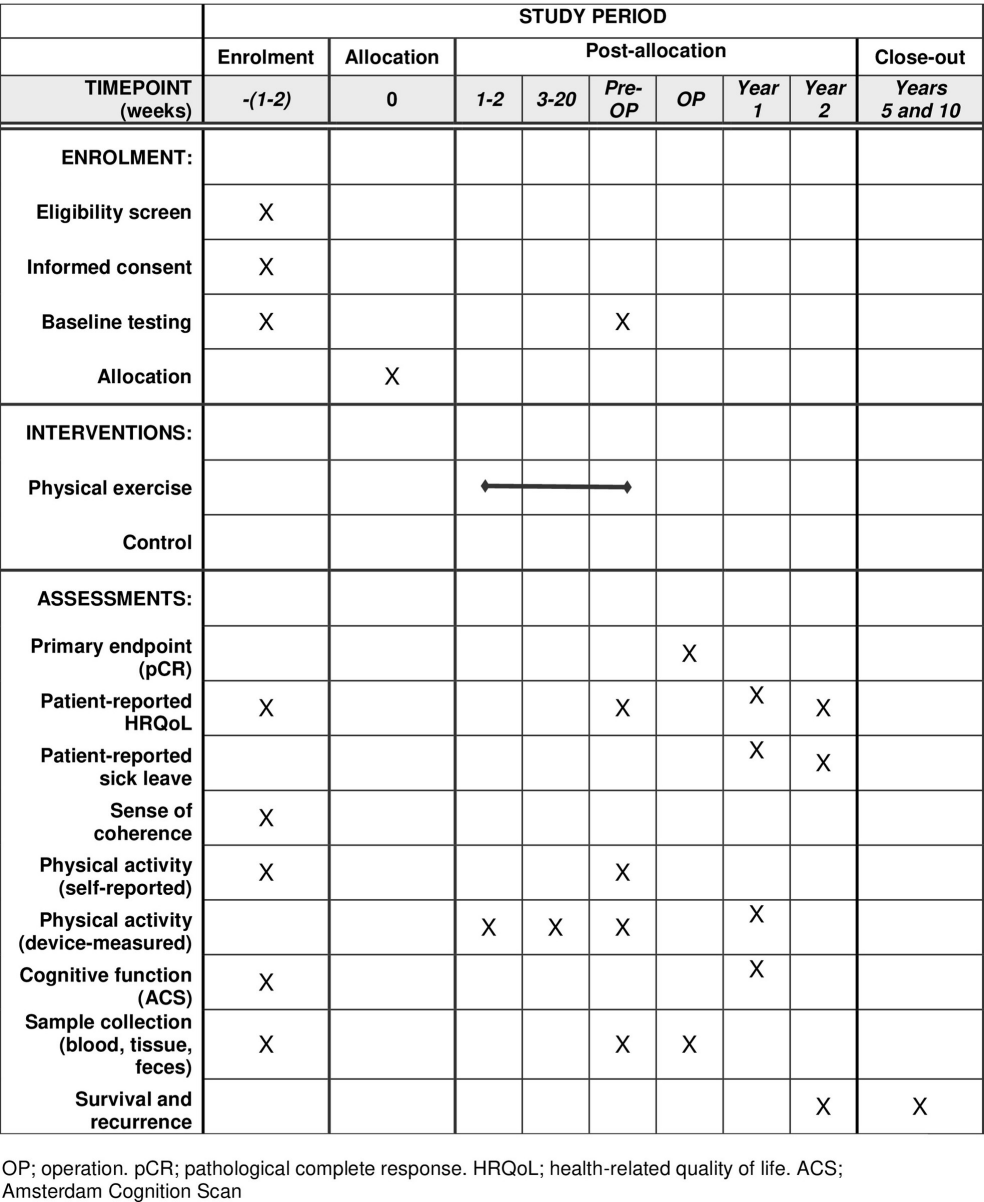
Schedule of enrolment, interventions, and assessments for the Neo-ACT trial.

**Fig 2:**
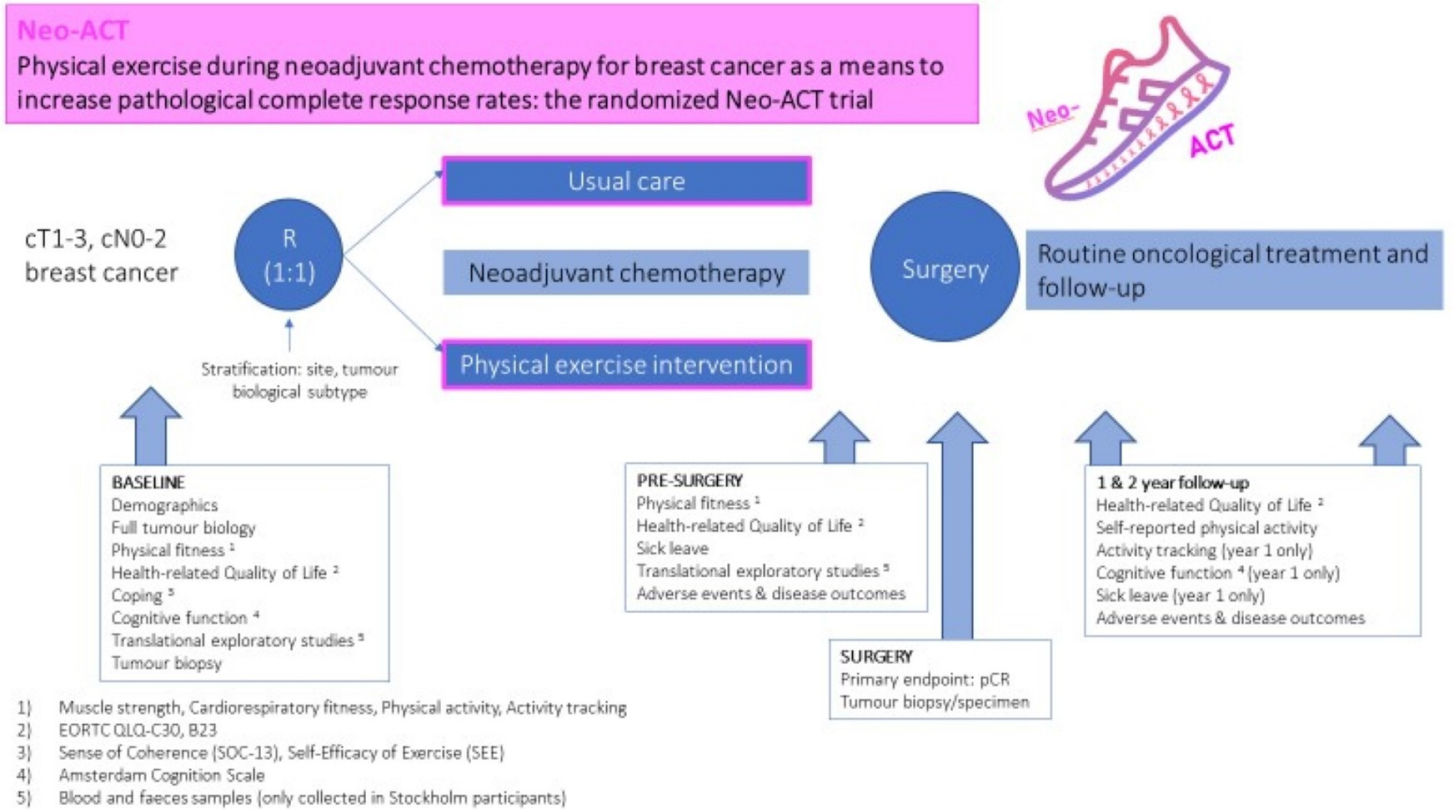
Schematic overview of the Neo-ACT trial.

Neo-ACT is a prospective randomized controlled multicentre trial, testing a home-based physical exercise intervention during NACT for BC (Figs 1 and 2). The primary endpoint is pCR, defined as ypT_0/is_N_0_. Secondary endpoints are patient-reported (health-related quality of life, self-reported physical activity), physiological (muscle strength, cardiorespiratory fitness, device-measured physical activity), toxicity-related (cognitive dysfunction, chemotherapy completion rates, unplanned hospital admissions, cardiac toxicity, sick leave) and oncological (Residual Cancer Burden, objective radiological tumour response according to RECIST criteria, recurrence-free, cancer-specific and overall survival at 2, 5 and 10 years). Furthermore, the trial will explore exercise-induced anti-tumoral mechanisms by hypothesis-generating translational analyses.

### Population

Clinically T1-3, N0-2 BC patients scheduled for NACT and surgery with curative intent while fulfilling all inclusion criteria and no exclusion criteria are eligible to participate in the trial (Fig 2 and Table 1). Patients with oligometastases amenable for such ablative treatment with curative intent are also eligible.

**Table 1:** Inclusion and exclusion criteria in the Neo-ACT trial.

Inclusion criteria	Exclusion criteria
• Patients with primary invasive breast cancer cT1-T3 cN0-2 • Full tumour biology available before initiation of NACT (ER, PR, HER2, tumour grade, and Ki67) • Written informed consent • Age ≥ 18 years	• Bilateral invasive breast cancer • Pregnancy or breast-feeding • Musculoskeletal, neurological, respiratory, metabolic or cardiovascular conditions that may prevent safe completion of the exercise and testing demands of the study • Currently performing equal to or more than 150 min of moderate to high intensity aerobic exercise including 2 sessions per week of moderate intensity resistance exercise

Informed consent is mandatory before randomization and will be obtained by physician or nurse with the appropriate delegation.

In case of premature abortion of NACT, participants may remain on the trial. Participants who have not received at least four three-weekly treatment cycles (i.e., twelve treatment weeks) of NACT will not be included in per-protocol analyses.

### Intervention

All participants undergo standardized tests of physical fitness and strength before start of NACT and after NACT but before surgery. Participants randomised to the exercise group complete two 60-min home-based exercise sessions per week from initiation of NACT to surgery (approx. five months). The program includes resistance and high-intensity interval training (HIIT). Patients are supported to exercise via the individualized exercise mobile application Vitala. Videos of variations of the resistance exercises and HIIT are included in the app, which allows each participant’s exercise program to be individually adapted relative to their health and fitness status both initially and throughout the trial. Participants are also contacted by their local physiotherapist/ exercise specialist remotely at least twice during the first four weeks. In addition, the local physiotherapist or exercise specialist has access to the mobile app’s interface and contacts the participant specifically in case no activity has been logged for one week. Weekly web-based support sessions offered by the coordinating trial committee are open to local physiotherapists or exercise specialists.

Sessions begin with a 3-min moderate intensity (12–13 on Borg’s Rate of Perceived Exertion (RPE) scale) warm-up. The first two weeks include only continuous moderate intensity aerobic exercise (15 min) as a gradual introduction to the exercise program. From week 3 onwards, the participants progress to HIIT which is 3 x 3-min intervals with 2 min of passive recovery in between. Participants can choose from a wide range of cardiorespiratory exercises suggested through Vitala e.g. high-intensity Tabata type activities, or stairclimbing. The physiotherapist will support the participants to increase the intensity of their exercise gradually during the duration of the intervention with an aim to reach 16–18 on the Borg’s scale. Participants are also asked to complete progressive resistance exercise from week 1 which includes ~7 exercises that target predominantly large muscle groups, e.g. chest, back, legs, biceps, triceps and abdominal muscles. Participants can choose which variation of the exercise to suit their preferences and to reach the intended intensity. The first two weeks, participants will aim to complete the resistance exercises at 60–70% of their 1-repetition maximum (1-RM), progressing to 70–80% 1-RM for the remaining weeks.

Altogether, patients are encouraged to accumulate at least 150 min of moderate physical activity each week, inclusive of the intervention exercise sessions.

### Control

The aim of the trial is to compare the effects of an exercise program with routine care rather than to test a specific type of exercise. Thus, the control group is a routine care control group, which means they may receive brief verbal or printed general information about the benefits of physical activity from the treating physicians or breast cancer nurses. Participation in voluntary training groups organised by the treating hospital is discouraged in order to not create cross-over effects between the groups. To measure actual physical activity in both groups, all participants will be wearing a Fitbit activity tracker so that any contamination in the control group can be accounted for in the analysis.

### Outcomes

The primary endpoint pCR is measured by histopathological assessment according to the TNM classification of the American Joint Committee on Cancer (ypT0/is ypN0) after breast and axillary surgery. A central review of histopathological tumour slides by an experienced breast pathologist blinded for assigned study arm is performed at Karolinska University Hospital in Stockholm for all included cases.

The secondary endpoints are:

1. Residual Cancer Burden (RCB), calculated using primary tumour bed area (mm x mm), overall cancer cellularity (%), percentage of cancer that is in situ disease (%), number of positive lymph nodes and diameter of largest nodal metastasis. Categories are RCB-0 (pCR), RCB-I, RCB-II and RC-III.
2. Objective tumour response according to RECIST criteria [23], measured as % change from largest radiological diameter of target lesion at baseline to pre-surgery (mammography or magnetic resonance tomography). No more than two target lesion are measured, i.e. the largest measurable lesions within the breast. Categories are: complete response (CR, disappearance of all target lesions), partial response (PR, at least a 30% decrease in the sum of the largest diameter (LD) of target lesions, taking as reference the baseline sum LD), progressive disease (PD, at least a 20% increase in the sum of the LD of target lesions, taking as reference the smallest sum LD recorded since the treatment started or the appearance of one or more new lesions) and stable disease (SD, neither sufficient shrinkage to qualify for PR nor sufficient increase to qualify for PD, taking as reference the smallest sum LD since the treatment started).
3. Overall, breast cancer-specific and recurrence-free survival at 2, 5 and 10 years. For overall survival, censuring is at death or at date for latest follow-up. For breast cancer-specific survival, censuring is at breast cancer-specific death or at date of last follow-up. For recurrence-free survival, censuring is at first local, regional or distant recurrence or at death or date of last follow-up. Contralateral invasive or in situ breast cancer or non-breast secondary malignancies are not counted as an event.
4. Health-related quality of life including a fatigue subscale, assessed by the EORTC QLQ-C30 and BR23 questionnaires at baseline, pre-surgery, and 1- and 2-year follow-up.
5. Self-reported physical activity assessed by the Modified Godin Leisure Time Physical activity questionnaire at baseline, pre-surgery, and 1- and 2-year follow-up.
6. Toxicity-related outcomes such as chemotherapy completion rates, number of unplanned hospital admissions during NACT, objective cognitive dysfunction measured by an online neuropsychological test (Amsterdam Cognition Scan) [24], cardiac toxicity (defined as either left ventricular ejection fraction (LVEF) decline >15% or LVEF decline below an absolute value of 50% or development of clinical heart failure), measured by echocardiogram at baseline and after 3 months of NACT in the HER2-positive subgroup, patient-reported percentage of sick leave pre-surgery and at 1- and 2-year follow-up, device-measured physical activity level assessed through a Fitbit activity tracker (baseline to 1-year follow-up), muscle strength assessed through the handgrip strength test and hypothetical 1-RM maximal leg muscle strength tests (baseline and pre-surgery), and estimated cardiorespiratory fitness assessed by the Ekblom-Bak submaximal cycle test (baseline and pre-surgery).

Attendance to the exercise sessions is continually monitored. Sense of coherence measured by the SOC-13 scale and the Self-Efficacy for Exercise measured by the SEE scale are used to better understand who adheres to the exercise intervention.

Participant demographics, disease and medical history, body mass index, age, sex, education level, and smoking status are initially recorded. Since this trial is not powered to assess survival and recurrence rates, follow-up ends two years after baseline. Register data will however be used in order to investigate overall and BC-specific survival rates as well as recurrence rates after 2, 5 and 10 years.

### Data management

Data are registered using an electronic Case Report Form (eCRF). Monitoring is performed according to Good Clinical Practice (GCP) guidelines. The eCRF provides data on age, initial tumour and lymph node characteristics deriving from clinical, radiological and histopathological assessment, details concerning type, dose and duration of neoadjuvant and adjuvant systemic therapy, as well as histopathological results at surgery and data on follow-up. Data are managed by the Clinical Trial Office at Centre for Clinical Cancer Studies, Karolinska University Hospital, Stockholm, Sweden. Recorded information is pseudonymised. The Neo-ACT trial is registered at www.clinicaltrials.gov (NCT05184582).

Ethical permission has been obtained by the Swedish Ethical Review Authority (Dnr 2022-02084-01). All participants must provide their written informed consent to participate in this trial.

### Follow-up

The trial ends for each participant followed for two years after the date of surgery, but also for participants who die, withdraw consent or are lost to follow-up. Follow-up can be conducted as telephone call or by post or E-mail, and also includes access to the participant’s medical file in order to check for survival and recurrence. Follow-up must be performed within +/- two months from the surgery date, and data are to be entered in the eCRF within one month from the follow-up date.

All participants, regardless of group assignment, are instructed to report the occurrence of exercise-related adverse events (AEs). The local investigator only documents AEs in relation to the intervention, which are exercise-related AEs requiring treatment or talking to a doctor or other health professional, causing any persistent worsening of participants’ health or well-being, new occurrence of substantial pain or swelling e.g. muscle tear, and occurring during or after the exercise session and requiring restrictions/alterations or early termination of the exercise, or any treatment or clarification by a physician. Local investigators make a severity assessment of the event according to the Common Terminology Criteria for Adverse Events Version 5 published November 27, 2017 [25]. AEs have to be reported as serious adverse event (SAE) only when they are related (possibly, probably, or definitely) to trial intervention. AEs related to NACT or adjuvant therapy are not considered as a SAE and are therefore exempted from expedited reporting. Trial intervention-related SAEs are documented and reported immediately (within a maximum of 24 hours).

### Estimated sample size and power

Patients are randomized in a 1:1 fashion. It is anticipated that the pCR rate will be approximately 30% in the control arm (all biological subgroups combined). We aim to increase the rate of pCR in the experimental arm to 40%, i.e. a 10 percentage points increase, which is regarded clinically relevant as it would translate into improved disease-related outcomes. With a power of 80% and an alpha of 5%, a total of 712 patients have to be included; 356 in each arm. Accounting for a drop-out rate of 10%, the trial aims to include 790 patients. Stratification at the moment of computerized randomization will be done based on site of treatment (hospital) and biological tumour subtype (ER+HER2-, ER+HER2+, ER-HER2+, ER-HER2-).

### Statistical analysis plan

All outcomes will primarily be analysed using an intention-to-treat approach, i.e. all trial participants will belong to the treatment group (intervention or control) they were assigned to, disregarding compliance. As sensitivity analysis, all outcomes will also be analysed using a per-protocol approach, meaning that participants in the intervention group who attend fewer than 65% of the prescribed physical exercise sessions will be excluded from analysis.

The dichotomous primary endpoint will be assessed through a multivariable logistic regression model, adjusting for biological tumour type. The treatment effect will be assessed in terms of the resulting odds ratio between the two treatment arms together with a 95% confidence interval and a complementary Wald test. A two-sided statistical test with 5% significance level will be used. Furthermore, differences in pCR rates between the two treatment arms will be explored for each biological tumour subtype. For the secondary outcomes, the differences between the treatment arms will be reported as an effect measure (e.g. odds ratio or mean difference) together with a complementary confidence interval and a two-sided statistical test of the effect parameter. A Bonferroni corrected significance level will be used.

### Independent safety analysis

A data monitoring committee consisting of three independent experts will perform a safety analysis with the purpose to assess the recruitment to the trial and the rate of adverse events, and to make sure that patients in the intervention group do not appear to fare significantly worse than patients in the standard of care group. The committee may recommend terminating the study if a significant benefit in favour of one group is shown, such that the HR for intervention versus standard of care significantly (p = 0.001) exceeds 1, if the recruitment is so low that that the necessary number of events is unlikely to be reached, or if there are serious concerns about unexpected AEs in the intervention group. If the committee determines that it is safe to proceed with the study, the results of the analysis will remain unknown to everyone except the committee members.

### Time plan

The Neo-ACT is registered at clinicaltrials.gov (NCT05184582) and opens for enrolment at several Swedish and Finnish sites from September 2022. Further participating sites will be added according to interest and need. Patient enrolment is estimated to be completed by the end of 2025, and first results on the primary endpoint are reported in 2026. A list of participating sites will be available at https://ki.se/mmk/neo-act-studien.

## Discussion

**Fig 3:**
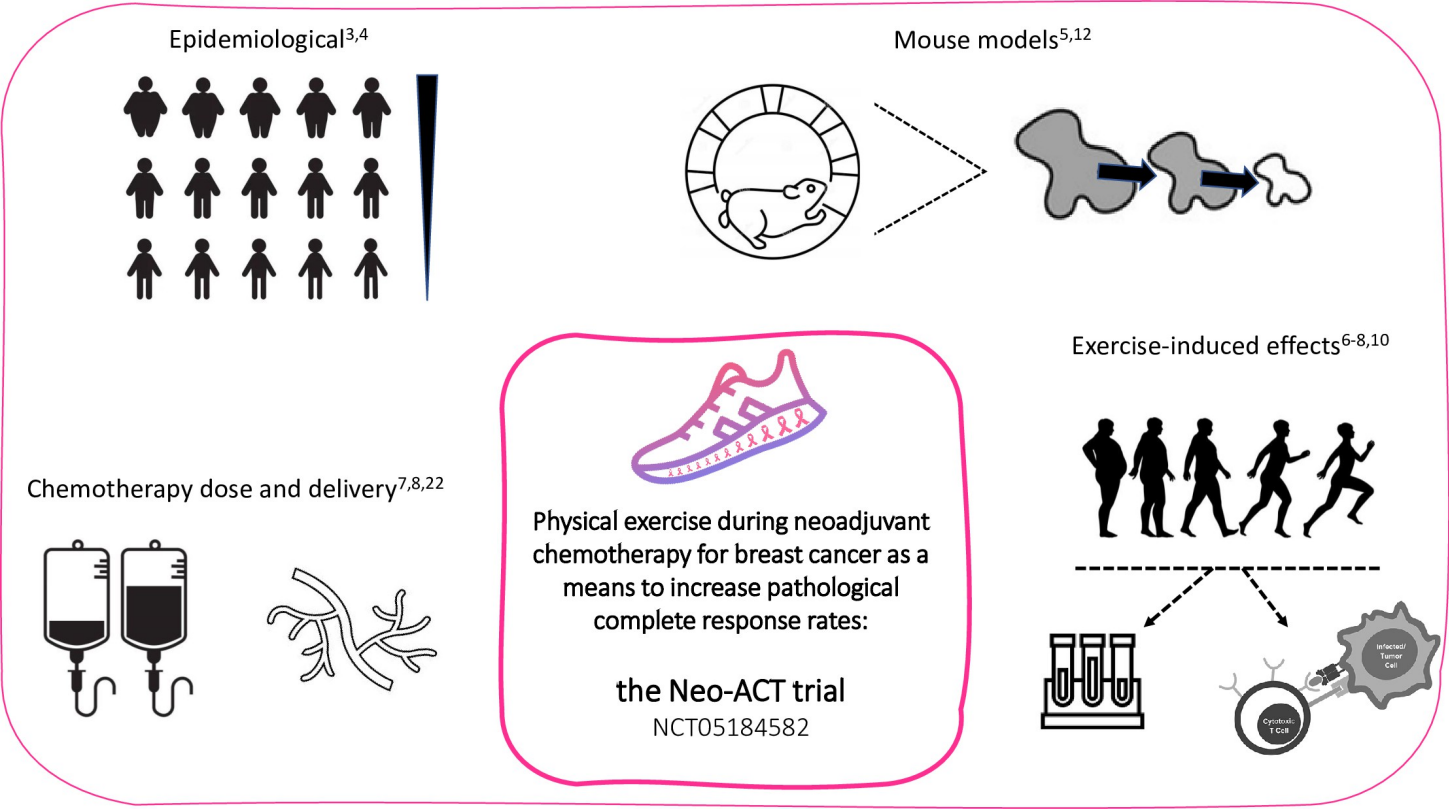
Schematic presentation of published evidence regarding physical activity and breast cancer.

There is mounting evidence for the anti-tumoral potential of physical exercise, which is the underlying rationale for the randomised Neo-ACT trial (Fig 3). If successful, this trial will lead to the implementation of physical exercise into NACT not only as an auxiliary means to reduce toxicity and improve well-being, but also as a therapeutic agent by itself.

Similar to neoadjuvant drug trials, sample calculation needs to define a clinically meaningful pCR difference to be achieved that could impact adjuvant treatment planning and in turn translate into more beneficial disease-related long-term outcomes. In the Neo-ACT trial, a 10% increase of pCR is estimated to be sufficiently relevant to view exercise as treatment rather than solely a supportive strategy. In comparison, the randomized Keynote 522 trial evaluating the addition of pembrolizumab to neoadjuvant chemotherapy for TNBC [26] and the KRISTINE trial [27], randomly assigning participants to trastuzumab emtansine plus pertuzumab or docetaxel, carboplatin, and trastuzumab plus pertuzumab, were designed to detect a pCR difference of 15%, but based on the already high pCR rates seen in TNBC and HER2-positive BC. The GeparOcto trial, which included luminal B subtypes only if lymph node positive, failed to show a proposed pCR difference of 10% assuming a pCR rate of 50% in the control group [28], and the GENEVIEVE trial was close to succeed in showing a proposed 10% (1.2 versus 10.8%) pCR improvement for TNBC or ER-positive/HER2-negative BC [29]. Importantly, a significant improvement of clinically relevant oncological secondary endpoints of the Neo-ACT trial such as Residual Cancer Burden, tumour shrinkage in accordance to RECIST criteria, or chemotherapy completion rates would still deliver a strong rationale to support integration of physical exercise into NACT schedules as standard practice, in addition to the improvement of patient-reported and toxicity-related outcomes.

In analogy to drug compliance, it is important for patients to adhere to the exercise prescription. Consequently, research has focused on strategies to enhance both attendance and adherence to exercise interventions. Technological support in the form of mobile apps is a potential sustainable strategy to improve attendance and adherence to exercise and rehabilitation programs [30]. While the effects of exercise on health are increased if the exercise program at least initially includes supervision [31], health care systems rarely have the resources to invite every patient undergoing NACT to supervised exercise programs. Another advantage of home-based exercise is that it significantly reduces time and travel burden for patients who often have frequent appointments. To increase feasibility and reach out to as many patients as possible, distance-based approaches to exercise participation must therefore be trialled. Finally, in this post-pandemic reality, possible future outbreaks of contagious diseases leading to (partial) lockdowns must be considered when developing effective, agile and flexible supportive healthcare strategies. Thus, it is vital that the proposed trial investigates innovative and potentially sustainable strategies to implement exercise programs for patients with cancer.

In some institutions in Sweden, physical exercise during NACT or adjuvant chemotherapy is already offered in voluntary training groups. This, and the fact that some international institutions already implement physical exercise as an integral part of their NACT strategy, underlines the general awareness of the benefit of physical exercise in the context of chemotherapy for BC. It is now of importance to evaluate the anti-tumoral effect of physical exercise beyond its symptom-relieving characteristics in order to gather strong evidence for the implementation of physical exercise into neoadjuvant and adjuvant strategies for all patients.

## Conclusion

In conclusion, accumulating data on the anti-tumoral effects of physical exercise create a rationale for testing physical exercise as a means to improve pCR rates in BC patients receiving NACT. While pCR rates are a reasonable surrogate endpoint for important long-term disease-related outcomes such as disease-free or cancer-specific survival in neoadjuvant intervention trials, Neo-ACT additionally collects data on important oncological secondary endpoints as well as toxicity-related and patient-reported endpoints, ensuring that any potential benefit of physical exercise in this context is covered and assessed. The integration of patient empowerment and involvement in treatment strategies is a crucial ingredient of personalised medicine.

## Supporting information

S1 File SPIRIT 2013 checklist: Recommended items to address in a clinical trial protocol and related documents*. (DOC)

S2 File (DOCX)
