# Supplementary material for: Physical exercise during neoadjuvant chemotherapy for breast cancer as a mean to increase pathological complete response rates: Trial protocol of the randomized Neo-ACT trial
Source: PLoS One. 2022 Oct 13;17(10):e0274804. doi: 10.1371/journal.pone.0274804 (PMC9562167; doi:10.1371/journal.pone.0274804)
Supplement: S2 File — (DOCX) [file pone.0274804.s002.docx]

**Physical exercise during neoadjuvant chemotherapy for breast cancer as a means to increase pathological complete response rates: the randomized Neo-ACT trial**

NCT05184582 (www.clinicaltrials.gov)

Full trial protocol version 1.1

Dated July 10, 2022

## Version identifier

Version 1.0, April 3, 2022

Version 1.1, July 10, 2022

Any relevant amendments to the protocol are first submitted to the responsible ethical committee and after approval disseminated to all participating sites.

## Sponsor

Karolinska Institutet, Dept. of Molecular Medicine and Surgery, 17176 Stockholm

## Principal investigators

**Jana de Boniface (overall coordinating investigator),** Associate Professor, Department of Molecular Medicine and Surgery, Karolinska Institutet, Stockholm, Sweden, and Department of Surgery, Capio St. Göran’s Hospital, Stockholm Sweden.

**Yvonne Wengström (coordinating investigator exercise)**, Professor, Department of Neurobiology, Care Sciences and Society, Karolinska Institutet, Stockholm, Sweden

**Renske Altena (coordinating investigator oncology/toxicity/translational research)**, PhD, Medical Unit Breast, Endocrine Tumours and Sarcoma, Theme Cancer, Karolinska University Hospital, Department of Oncology Pathology, Karolinska Institutet, Stockholm, Sweden

**Cecilia Haddad Ringborg** **(coordinator exercise),** PhD, Department of Neurobiology, Care Sciences and Society, Karolinska Institutet, Stockholm, Sweden

## International collaboration

**Peeter Karihtala (national coordinator Finland)**, Professor, Research Director at Helsinki University Hospital, Comprehensive Cancer Center, Finland

## Advisor Pathology

**Johan Hartman**, Professor, Karolinska Institutet, Dept. of Oncology-Pathology

## Advisor Radiology

**Sophia Zackrisson,** Professor, Skåne University Hospital Malmö and Lund University

## Collaborators translational projects

**Johan Hartman**, Professor, Karolinska Institutet, Dept. of Oncology-Pathology

**Theodoros Foukakis**, Ass. Prof., Karolinska Institutet, Dept. of Oncology-Pathology

**Dhifaf Sarhan**, Associate Professor, Karolinska Institutet, Dept of Laboratory Medicine

**Barbro Linderholm**, Professor, Sahlgrenska University Hospital, Dept. of Oncology

## Trial statistician

**Robert Szulkin,** PhD, Scandinavian Development Services, Danderyd, Sweden

## Patient representative

**Åsa Magnusson**, Swedish Breast Cancer Association (Bröstcancerförbundet)

## Trial Coordinating Center

Clinical Trial Office (CTO)

Centre for Clinical Cancer Studies (CKC)

Theme Cancer

Karolinska University Hospital

17176 Stockholm

Project manager: Natalia Pohl, [natalia.pohl@regionstockholm.se](mailto:natalia.pohl@regionstockholm.se)

Monitor, Sweden: Yvonne Larsén, [yvonne.larsen@regionstockholm.se](mailto:yvonne.larsen@regionstockholm.se)

## Data management

**Mats Hellström**, data manager, Clinical Trials Office, Karolinska University Hospital, Stockholm, Sweden

Table of Contents

[Version identifier 2](#_Toc110673342)

[Sponsor 2](#_Toc110673343)

[Principal investigators 2](#_Toc110673344)

[International collaboration 2](#_Toc110673345)

[Advisor Pathology 2](#_Toc110673346)

[Advisor Radiology 2](#_Toc110673347)

[Collaborators translational projects 3](#_Toc110673348)

[Trial statistician 3](#_Toc110673349)

[Patient representative 3](#_Toc110673350)

[Trial Coordinating Center 3](#_Toc110673351)

[Data management 3](#_Toc110673352)

[Synopsis 6](#_Toc110673353)

[Background 10](#_Toc110673354)

[Purpose and aims 12](#_Toc110673355)

[Hypotheses 13](#_Toc110673356)

[Method 13](#_Toc110673357)

[Study design 13](#_Toc110673358)

[Population 14](#_Toc110673359)

[Intervention 14](#_Toc110673360)

[Control 16](#_Toc110673361)

[Outcomes, Variables and measures 17](#_Toc110673362)

[Study calendar 19](#_Toc110673363)

[Data management 20](#_Toc110673364)

[Monitoring and follow-up 20](#_Toc110673365)

[End of trial 21](#_Toc110673366)

[Adverse Events 21](#_Toc110673367)

[Estimated sample size and power 23](#_Toc110673368)

[Statistical analysis plan 23](#_Toc110673369)

[Ethical considerations 29](#_Toc110673370)

[Withdrawal 29](#_Toc110673371)

[Publication policy 29](#_Toc110673372)

[Time plan 30](#_Toc110673373)

[Translational research 30](#_Toc110673374)

[Faeces microbiome 30](#_Toc110673375)

[Intratumoral and circulating Natural Killer (NK) cells 31](#_Toc110673376)

[Tumour gene profiling 32](#_Toc110673377)

[Tumour microenvironment 32](#_Toc110673378)

[Circulating tumour cells and tumour DNA 33](#_Toc110673379)

[The effects of human adipocytes on breast cancer progression and metastasis 33](#_Toc110673380)

[References 35](#_Toc110673381)

# Synopsis

| **Title** | Physical exercise during neoadjuvant chemotherapy for breast cancer as a means to increase pathological complete response rates |
| --- | --- |
| **Short title** | Neo-ACT trial (NCT05184582) |
| **Trial design** | Prospective randomized clinical trial |
| **Trial rationale** | Neoadjuvant chemotherapy (NACT) is the current standard of care for patients with breast tumours larger than 20 mm and/or lymph node metastases, particularly Human Epidermal growth factor Receptor 2 (HER2) and triple negative breast cancer (TNBC). The best proof of NACT efficacy is pathological complete response (pCR), i.e. the absence of residual invasive tumour in the breast and the axillary lymph nodes. Today, pCR is frequently used as surrogate endpoint in oncological pharmaceutical trials focusing on new compounds often in combination with the current standard of care. While NACT has the advantage to offer fast-track approval of new drug compounds in oncology, early expectations of improved survival rates have not been met. Additional systemic drug regimens come, however, at a cost, and associated side effects and toxicities are important to bear in mind. It is therefore utterly compelling to conceive that improved NACT efficacy – and thus de-escalated locoregional therapy – may be achieved by a non-toxic patient-driven life-style intervention such as physical exercise.  Being physically active reduces the risk for breast cancer by 20-30%, and exercise induces reductions of tumour growth in animal models of breast cancer. Exercise may act through reduced systemic inflammation and enhanced anti-tumoural immune cell function, improve blood flow and perfusion and thus tumour susceptibility to systemic treatment, reduce systemic inflammation and enhance immune cell functions. The short-term stress of a single bout of physical exercise in healthy human subjects can induce a release of immune cells into the circulation, and primary tumour growth is reduced in mice exposed to voluntary running.  Physical exercise during chemotherapy is feasible and safe. Observational studies show that exercise in breast cancer has a protective effect regarding recurrence and mortality. Long-term follow-up of aerobic and resistance exercise shows enhanced survival in patients with breast cancer. In the randomized OptiTrain trial, resistance and high-intensity interval training (HIIT) during postoperative chemotherapy had positive effects on fatigue and muscle strength, muscle mass and function. Chemotherapy completion rates are improved by exercise. Exercise may thus result in improved pCR rates through systemic anti-inflammatory effects and improved chemotherapy completion rates given at full dosage due to the favourable effects of exercise on treatment tolerability. |
| **Endpoints** | The **primary** endpoint is pathological complete response (pCR).  The **secondary** endpoints are:  - Residual Cancer Burden (RCB)  - Objective tumour response (RECIST)  - All-cause, breast cancer-specific, and recurrence-free survival at 2, 5 and 10 years  - Health-related quality of life assessed by the EORTC QLQ-C30 and BR23 questionnaires  - Self-reported physical activity (Modified Godin Leisure Time Physical activity questionnaire)  - Toxicity-related outcomes (chemotherapy completion rates, number of unplanned hospital admissions during NACT, objective cognitive dysfunction (Amsterdam Cognition Scale), cardiac toxicity and sick leave)  - Device-measured physical activity level (Fitbit activity tracker)  - Muscle strength (handgrip strength test and hypothetical 1-RM maximal leg muscle strength tests)  - Cardiorespiratory fitness (Åstrand submaximal cycle test) |
| **Patient selection** | Clinically T1-3, N0-2 breast cancer patients scheduled for NACT and surgery with curative intent.  **Inclusion criteria**:   - Patients with primary invasive breast cancer cT1-T3 cN0-2 - Full tumour biology (ER, PR, HER2, tumour grade, and Ki67) available before initiation of NACT - Oral and written consent - Age ≥ 18 years   **Exclusion criteria**   - Bilateral invasive breast cancer - Pregnancy or breast-feeding - The presence of musculoskeletal, neurological, respiratory, metabolic or cardiovascular conditions that may prevent safe completion of the exercise and testing demands of the trial - Currently performing equal to or more than 150 mins of moderate to high intensity aerobic exercise and 2 sessions per week of moderate intensity resistance exercise |
| **Intervention** | Participants randomized to the exercise group will complete two home-based 60-min exercise sessions per week from initiation of NACT to surgery (approx. five months):   - Progressive home exercise program by an individualised mobile phone application, supported by local physiotherapists and by online meetings - Initial exercise intensity individually tailored to each patient’s fitness at baseline and rate of perceived exertion during the program and adapted if required - Sessions will begin with a 3-minute moderate intensity (12-13 on Borg’s Rate of Perceived Exertion (RPE) scale) warm-up.   First two weeks: continuous moderate intensity aerobic exercise (15 mins) at 14-15 RPE before starting interval training. From week 3, high-intensity interval training, i.e. 3x3-minute intervals at RPE 16-18 e.g. on a cycle ergometer and high-intensity Tabata type activities, running, or stairclimbing with 2 minutes of passive or active recovery in between bouts.  Moderate intensity progressive resistance exercises include ~7 exercises e.g. chest and leg press, seated row, bicep curl, triceps press, leg curl, and abdominal exercises.  In total, patients will be encouraged to accumulate 150 minutes of physical activity each week.  No exercise within 48 hours of chemotherapy administration. |
| **Control** | Routine information on benefit of physical exercise as per clinical guidelines and local practice. |
| **Follow-up** | Each patient is followed up for two years after surgery regarding secondary endpoints. Survival and recurrence data are obtained via national registers at 5 and 10 years. |
| **Statistical considerations** | Patients will be randomized in a 1:1 fashion. In order to detect an increase of the pCR rate in the experimental arm by 10%, which is regarded clinically relevant, and using a power of 80% and an alpha of 5%, a total of 712 patients have to be included; 356 in each arm. Accounting for a drop-out of 10%, the trial will include 790 patients. Stratification at the moment of computerized randomization will be based on hospital and biological tumour subtype (ER+HER2-, ER+HER2+, ER-HER2+, ER-HER2-). |
| **Time plan** | Trial initiation: September 2022  Enrolment phase: September 2022 – December 2025  Reporting of primary endpoint: September 2026 |

# Background

***Use of neoadjuvant (preoperative) chemotherapy in breast cancer***

Neoadjuvant chemotherapy (NACT) has a history of being reserved for non-operable, locally advanced breast cancer (BC) but is increasingly used for patients with early BC. It is strongly recommended by national^1^ and international^2^ guidelines especially in the triple-negative (TNBC) and HER2-positive BC subtypes, and in case of lymph node metastases. In Sweden, 14% of all newly diagnosed early BC patients suitable for surgery (1041 out of 7289 individuals according to the National Breast Cancer Register) received NACT in 2019 (22% in Stockholm), while this figure was 35% (Stockholm 45%) for TNBC and HER2-positive BC, and 50% (Stockholm 62%) for all clinically node-positive BC. Combining lymph node metastases and tumour subtype, the rate of NACT was as high as 68% (Stockholm 80%) in clinically node-positive TNBC and HER2-positive BC in 2019.

The best proof of NACT efficacy is pathological complete response (pCR), i.e. the absence of residual invasive tumour in the breast and the axillary lymph nodes. Especially in the above-mentioned tumour subtypes TNBC and HER2-positive breast cancer, published pCR rates are high (50.7% in TNBC and 63.7% in HER2-positive, oestrogen receptor (ER)-negative BC) and strongly predict improved survival^3^. In luminal BC (ER-positive, HER2-negative), published pCR rates are substantially lower (10.8% if HER2 negative, 29.4% if HER2 positive)^3^ which underlines the need for novel regimens including anti-endocrine strategies. Today, pCR is frequently used as surrogate endpoint in oncological pharmaceutical trials focusing on new compounds often in combination with the current standard of care (traditionally chemotherapy and/or targeted therapies with anti-HER2 and anti-ER drugs, but now also immunotherapy, CDK4/6 inhibitors and drug-antibody conjugates).

***Need to improve pCR rates***

While NACT has the advantage to offer fast-track approval of new drug compounds in oncology, such as the FDA approval of pertuzumab after the Neosphere trial published first in 2012^4^, early expectations of improved survival rates simply by reversing the traditional order of treatments (surgery followed by adjuvant systemic therapy) have not been met^5^. Only recently, however, the Keynote 522 trial could show both a higher pCR rate and an improved event-free survival in TNBC patients receiving the anti-PD1 antibody pembrolizumab, which fuels hopes that specific subgroups will be identified who gain a survival benefit from NACT when compared to standard treatment^6^. Apart from improving outcomes in specific subgroups, NACT facilitates research into post-neoadjuvant systemic treatment strategies, which has already resulted in supplemental therapies such as adjuvant capecitabine in HER2-negative BC^7^ and T-DM1 in HER2-positive BC^8^. It also allows for less extensive surgery by tumour shrinkage and conversion of node-positive BC into node-negative disease, which may be of great benefit for the individual patient in terms of postoperative morbidity and quality of life. Additional systemic drug regimens come, however, at a cost, and associated side effects and toxicities are important to bear in mind. It is therefore utterly compelling to conceive that improved NACT efficacy – and thus de-escalated locoregional therapy – may be achieved by a non-toxic patient-driven life-style intervention such as physical exercise. Such patient empowerment is especially important in the view of the common clinical observation that most patients request to know how they themselves may contribute to a favourable course of their disease and treatment.

***Evidence on physical exercise and cancer***

Epidemiological research shows that being physically active reduces the risk for breast cancer by 20-30%^9^ and has a similarly protective effect in women carrying high-risk BC mutations such as BRCA1 and BRCA2^10^. Since the 1960s, numerous intervention studies report exercise-induced reductions of tumour growth in animal models of breast cancer^11^. Mechanistically oriented preclinical trials suggest that exercise act through reduced systemic inflammation and enhanced anti-tumoural immune cell function^12,13^. Other studies show an altered phenotype of tumour vasculature with exercise, improving blood flow and perfusion, making the tumour more susceptible to systemic treatment^12,13^. Further suggested mechanisms for the anti-tumoural effects of exercise include weight control, endocrine effects, less systemic inflammation (reflected by lower CRP and pro-inflammatory cytokine levels in serum), improved immune cell functions such as increased recruitment and cytotoxic activity of CD8+ T-cells and NK cells, and a shift towards an anti-tumorigenic (Th1/M1) profile^12-14^. In the tumour microenvironment, the level of inflammatory cell infiltration increases markedly in response to physical exercise^15^. Here, immune cells can provide anti-tumour immune responses and thus improve survival outcomes, or instead facilitate tumour growth and metastasis. Infiltrating cytotoxic CD8+ T-cells and NK cells predict a favourable clinical outcome in several solid human cancers, including breast cancer; in contrast, high levels of infiltrating T-regulatory cells and myeloid cells are linked to tumour progression and poor prognosis^15^. The short-term stress of a single bout of physical exercise in healthy human subjects can induce a release of immune cells such as granulocytes, monocytes and NK cells, as well as CD4- and CD8-positive T-cells^16^ into the circulation. In a recent study, primary tumour growth was reduced in multiple murine tumour models exposed to voluntary running^17^. This was attributed to enhanced tumour infiltration of NK cells, activated by an increase in systemic levels of epinephrine during exercise^18^. Very recently, a non-randomised prospective trial evaluated physical exercise during neoadjuvant treatment in patients with oesophageal cancer; participants in the exercise group had significantly more tumour regression at surgery than those in the control group^19^.

***Physical exercise and chemotherapy***

Physical exercise during chemotherapy is deemed feasible and safe^20^, even when performed via tailored home-based exercise during neoadjuvant chemotherapy^21^. We have evidence from observational studies showing that exercise following a BC diagnosis has a protective effect regarding recurrence, all-cause and cancer-specific mortality^22^. Furthermore, long-term follow-up of a randomized exercise intervention consisting of aerobic and resistance exercise showed enhanced recurrence-free and overall survival in patients with breast cancer^23,24^. In the randomized OptiTrain trial, resistance and high-intensity interval training (HIIT) during postoperative chemotherapy had positive effects on fatigue and muscle strength^25^, muscle mass and function^26^.

Chemotherapy completion at full dosage is strongly associated with an improved prognosis^27^, but dose reductions occur in at least a third of BC patients. Importantly, chemotherapy completion rates can be improved by an exercise program of combined resistance and aerobic training^28^. Physical exercise may thus result in improved pCR rates after NACT not only through proposed systemic anti-inflammatory effects, but also through improved chemotherapy completion rates given at full dosage due to the favourable effects of exercise on fatigue, muscle strength and cardiorespiratory fitness which drive improvements in treatment tolerability. Thus, there is great potential for physical exercise to be put forward as a feasible and effective strategy to support patients to tolerate treatments, which needs to be corroborated in prospective trials.

Physical exercise during NACT and its effects on treatment response and on pCR rates has never been tested. According to clinicaltrials.gov (searched March 22, 2022), there are only two open randomized physical exercise trials in neoadjuvant treatment of BC with oncological outcomes, namely the BENEFIT trial (Germany, N=120) and the Neo-Train trial (Denmark, N=100). Both small trials have the reduction of tumour size through NACT as their primary endpoint. Another trial is registered but not yet recruiting, the NEOLIFE trial (no oncological endpoint, NCT04135586). The Neo-ACT trial goes one step further and explores physical exercise as a means to improve oncological outcomes on a clinically relevant scale.

# Purpose and aims

The Neo-ACT trial is a prospective randomized controlled multicentre trial testing the effect of a physical exercise intervention during neoadjuvant chemotherapy (NACT) on the primary endpoint pathological complete response (pCR). Secondary aims are patient-related outcomes (health-related quality of life, self-reported physical activity), physiological outcomes (muscle strength, cardiorespiratory fitness, device-measured physical activity), and toxicity-related outcomes (cognitive dysfunction, chemotherapy completion rates, unplanned hospital admissions, cardiac toxicity, sick leave). Furthermore, the trial will explore how physical exercise affects anti-tumoral mechanisms inherent to therapy or host by hypothesis-generating translational analyses in a patient subset.

# Hypotheses

1. A physical exercise intervention improves pCR rates by 10% in the intervention group as compared with the control group.

2. A physical exercise intervention improves secondary outcomes such as patient-related outcomes (health-related quality of life, self-reported physical activity), physiological outcomes (muscle strength, cardiorespiratory fitness, device-measured physical activity), and toxicity-related outcomes (cognitive dysfunction, chemotherapy completion rates, unplanned hospital admissions, cardiac toxicity, sick leave).

Translational hypotheses will be tested in a subpopulation and will address which biological factors and mechanisms, assessed by contemporary translational studies from blood, faeces and tissue samples, are involved in anti-tumoral effects induced by physical exercise.

# Method

## Study design

The Neo-ACT trial is a prospective randomized trial with the primary endpoint pathological complete response (pCR) after neoadjuvant chemotherapy (NACT) for breast cancer (BC).


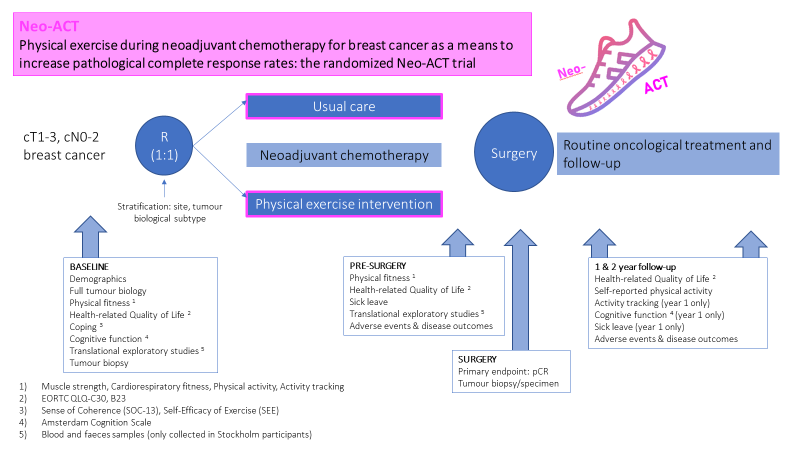


## Population

Clinically T1-3, N0-2 BC patients scheduled for NACT and surgery with curative intent and fulfilling all inclusion criteria and no exclusion criteria as listed below. Patients with oligometastases scheduled for such curative treatment are eligible. Baseline stratification is performed based on treating hospital and biological tumour subtype (ER+HER2-, ER+HER2+, ER-HER2+, ER-HER2-) prior to randomization.

Eligible patients are identified at pre-NACT multidisciplinary team conferences and then receive information about the trial by their oncologist or surgeon, avoiding undue delays. Informed consent is mandatory before randomization and can be obtained by physician or nurse with the appropriate delegation. All participants will undergo standardised tests of physical condition and strength before start of NACT and after NACT but before surgery.

| **Inclusion criteria** | **Exclusion criteria** |
| --- | --- |
| - Patients with primary invasive breast cancer cT1-T3 cN0-2 - Full tumour biology available before initiation of NACT (ER, PR, HER2, tumour grade, and Ki67) - Written informed consent - Age ≥ 18 years | - Bilateral invasive breast cancer - Pregnancy or breast-feeding - The presence of musculoskeletal, neurological, respiratory, metabolic or cardiovascular conditions that may prevent safe completion of the exercise and testing demands of the study - Currently performing equal to or more than 150 mins of moderate to high intensity aerobic exercise and 2 sessions per week of moderate intensity resistance exercise (WHO criteria) |

In case of pre-term abortion of NACT, participants may remain on the trial. Participants who have not received at least four treatment cycles (three weekly treatments of e.g. paclitaxel correspond to one treatment cycle) as NACT are excluded from per-protocol analyses (see statistical analysis plan).

### Intervention

Participants randomized to the exercise group will complete two 60-min home-based exercise sessions per week from initiation of NACT to surgery (approx. five months) via a mobile application. To digitalise exercise interventions:

- Patients will receive instructions by an exercise physiologist or physiotherapist and perform training via the individualised training mobile application Vitala, supported by contact with a local physiotherapist/physiologist and remotely via zoom.
- The Vitala mobile phone application provides exercise instructions and support and measures program adherence and symptom reporting. Videos of variations of the resistance training and high-intensity interval training (HIIT) exercises are included in the app and are individually adapted to each participant.
- Initial exercise intensity will be individually tailored to each patient’s fitness at baseline and rate of perceived exertion during the program and adapted if required
- Sessions will begin with a 3-minute moderate intensity (12-13 on Borg’s Rate of Perceived Exertion (RPE) scale) warm-up.

#### Aerobic exercise component

The first two weeks will include continuous moderate intensity aerobic exercise (15 mins) at 14-15 RPE to familiarise the patients with the exercise program before starting the interval training. From week 3 onwards, the patients will progress to high-intensity interval training which will be 3 x 3-minute intervals at RPE 16-18, e.g. completed on a cycle ergometer or by high-intensity Tabata type activities, running, or stairclimbing with 2 minutes of passive or active recovery in between bouts.

#### Resistance exercise component

Moderate intensity progressive resistance exercises will include ~7 exercises e.g. chest and leg press, seated row, bicep curl, triceps press, leg curl, and abdominal exercises performed as home-based exercises. In addition, patients will be encouraged to accumulate 150 minutes of physical activity each week (inclusive of the exercise in the structured exercise sessions).

#### Compliance: Supervised versus home-based exercise

In analogy to drug compliance, it is important for patients to adhere to the exercise prescription. Consequently, research has focused on strategies to enhance both attendance and adherence to exercise interventions. Technological support in the form of mobile apps is a potential sustainable strategy to improve attendance and adherence to exercise and rehabilitation programs^29^. On the other hand, the effects of exercise on health are increased if the exercise program at least initially includes supervision^30^. Health care systems unfortunately rarely have the resources to invite every patient undergoing NACT to supervised weekly exercises. To increase feasibility and reach out to as many patients as possible, distance-based approaches must therefore be included. Another advantage of home-based exercise is that it significantly reduces time and travel burden for patients who often have frequent appointments. Thus, it is vital that the proposed trial investigates innovative and potentially sustainable strategies to implement exercise programs for patients with cancer.

#### The mobile application

Participants randomised to the intervention group will receive instructions to download and use the training Vitala mobile application. Vitala has been developed to support patients with various physical limitations and diseases in need of medical exercise programs, in both rural and urban areas, and help them to independently create and maintain exercise routines.

The main features of the Vitala app include a Medical Exercise Generator specifically adapted to this trial and in-app self-monitoring services. Before using the app, participants receive instructions and guidance on how to set up and use the app, and fill out an in-app health questionnaire to ensure that all of Vitala’s functionalities and features are custom-tailored for each user.

The Vitala app has been developed in co-creation with patients and a cross-disciplinary research team consisting of researchers in physiotherapy, medicine, informatics and computer science, including experts in e-health. The app provides a large repository of evidence-based exercises in video formats and allows the user (in this case the trial team) to independently create an individualized medical exercise program based on preferences, disease, perceived energy levels and functional limitations. To help the patient to adhere to the program, the app offers feedback on exercise pattern, access to self-monitoring and possibility to submit questions regarding the program.

### Control

The aim of the trial is to compare the effects of an exercise program with routine care rather than to test a specific type of exercise. Thus, the control group is a routine care control group, which commonly implies brief verbal, general information about the benefits of physical activity from the treating physicians or breast cancer nurses. Participation in voluntary training groups organised by the treating hospital is not encouraged for participants since this implies that physical activity is potentially increased to the level specified in exclusion criteria. It is important to acknowledge that individuals consenting to an exercise trial may be more predisposed to exercising and may thus continue performing physical exercise if they are allocated to the control group. To measure this potential effect, all physical activity of both groups will be digitally collected so any “contamination” in the control group can be accounted for in the analysis. Importantly, the trial pursues an active recruitment strategy in order to meet potential participants at their respective fitness level however little they may be used to exercise. The control group will not have access to trial-specific exercises via the mobile application.

##

## Outcomes, Variables and measures

The **primary endpoint** pCR is measured by histopathological assessment according to the TNM classification of the American Joint Committee on Cancer (ypT0/is ypN0) after breast and axillary surgery (approx. 5 months after initiation of NACT, according to standard of care).

In order to ensure congruence in assessment of pCR between study sites, a central review of histopathological tumour slides will be performed for all included cases. Routine slides will be collected from participating sites and digitally scanned for central pathology review at Karolinska University Hospital in Stockholm. Slides will thereafter be returned to the study sites.

The **secondary** endpoints are:

1. Residual Cancer Burden (RCB), calculated using primary tumour bed area (mm x mm), overall cancer cellularity (%), percentage of cancer that is in situ disease (%), number of positive lymph nodes and diameter of largest nodal metastasis (<http://www3.mdanderson.org/app/medcalc/index.cfm?pagename=jsconvert3>). Categories are RCB-0 (pCR), RCB-I, RCB-II and RC-III.
2. Objective tumour response according to RECIST criteria^31^, measured as % change from largest radiological diameter of target lesion at baseline to pre-surgery (mammography or magnetic resonance tomography). No more than two target lesion are measured, which are the largest measurable lesions within the breast. Categories are: complete response (CR, disappearance of all target lesions), partial response (PR, at least a 30% decrease in the sum of the largest diameter (LD) of target lesions, taking as reference the baseline sum LD), progressive disease (PD, at least a 20% increase in the sum of the LD of target lesions, taking as reference the smallest sum LD recorded since the treatment started or the appearance of one or more new lesions) and stable disease (SD, neither sufficient shrinkage to qualify for PR nor sufficient increase to qualify for PD, taking as reference the smallest sum LD since the treatment started).
3. Overall, breast cancer-specific and recurrence-free survival at 2, 5 and 10 years. For overall survival, censuring is at death or at date for latest follow-up. For breast cancer-specific survival, censuring is at breast cancer-specific death or at date of last follow-up. For recurrence-free survival, censuring is at first local, regional or distant recurrence or at death or date of last follow-up. Contralateral invasive or in situ breast cancer or non-breast secondary malignancies are not counted as an event, in accordance with STEEP criteria^32^.
4. Health-related quality of life including fatigue, assessed by the EORTC QLQ-C30 and BR23 questionnaires (baseline, pre-surgery, 1- and 2-year follow-up)
5. Self-reported physical activity (Modified Godin Leisure Time Physical activity questionnaire) at baseline, pre-surgery, and 1- and 2-year follow-up, facilitating subsequent adjustment for any cross-contamination in the control group
6. Toxicity-related outcomes:
   1. Chemotherapy completion rates, i.e. the proportion of participants receiving the planned number of treatments at full dosage regarding neoadjuvant chemotherapy (measured pre-surgery).
   2. Number of unplanned hospital admissions during neoadjuvant chemotherapy.
   3. Objective cognitive dysfunction measured by an online neuropsychological test (Amsterdam Cognition Scan)^33^ (baseline and 1-year follow-up).
   4. Cardiac toxicity (defined as either left ventricular ejection fraction (LVEF) decline >15% or LVEF decline below an absolute value of 50% or clinical heart failure), to be measured by echocardiogram at baseline and after 3 months of NACT in the HER2-positive subgroup
   5. Sick leave (patient-reported percentage of sick leave as a single measurement pre-surgery and at 1- and 2-year follow-up)
7. Device-measured physical activity level and resting heart rate assessed through the Fitbit activity tracker (baseline to 1-year follow-up).
8. Muscle strength assessed through the handgrip strength test and hypothetical 1-RM maximal leg muscle strength tests (baseline and pre-surgery).
9. Cardiorespiratory fitness assessed by the Ekblom-Bak submaximal cycle test (baseline and pre-surgery).

#### Additional measures

Attendance to the exercise sessions and adherence to the exercise prescription will be continually monitored and recorded. Sense of coherence measured by the SOC-13 scale and the Swedish version of the original Self-Efficacy for Exercise (SEE) scale will be used to better understand who adheres to and attends the exercise intervention.

Participant demographics, disease and medical history, body mass index, age, sex, education level, and smoking status will be recorded. Since this trial is not powered to assess survival and recurrence rates since follow-up will end two years after baseline. Register data will however be used in order to investigate overall and BC-specific survival rates after 2, 5 and 10 years.

## Study calendar

| ***Time point*** |  | |
| --- | --- | --- |
| Baseline (before initiation of NACT) | - Verify eligibility - Informed consent - Three 14G tumour biopsies in addition to diagnostic biopsy* - Blood and plasma samples (4-5 x 10 ml) * - Faeces samples * - Full tumour biology (ER, PR, HER2, tumour grade, and Ki67)   Baseline questionnaires and testing   1. EORTC QLQ-C30 (quality of life) 2. EORTC QLQ-B23 (quality of life, breast cancer) 3. Sense of coherence (SOC-13) 4. Godin Leisure Time Self-Reported Physical Activity questionnaire 5. Self-Efficacy of Exercise (SEE) questionnaire 6. Hand grip muscle strength test 7. 1-RM leg press test 8. Ekblom-Bak submaximal cardiorespiratory cycle test 9. Amsterdam Cognition Scan 10. BMI, body fat composition, haemoglobin levels, blood pressure, demographics and medical history | |
| **Randomization** | ***Standard*: Usual care**  ***Intervention*: Physical exercise intervention** | |
| Pre-surgery | Repeat baseline questionnaires and testing (1.-2., 4.-8., 10.)  Device-measured physical activity (Fitbit activity tracker)  Blood (4-5 x 10 ml) and faeces samples *.  Record toxicity-related outcomes and treatment details (CRF) | |
| Surgery | Breast and axillary surgery: endpoint pCR assessed and recorded.  Tumour tissue collected and stored at trial biobank* | |
| Postoperative treatment | Adjuvant treatment as per clinical routine | |
| **Follow-up (years after surgery)** | **1** | **2** |
| EORTC QLQ-C30* | x | x |
| EORTC QLQ-B23* | x | x |
| Record recurrence and survival | x | x |
| Godin (physical activity)* | x | x |
| SEE scale | x |  |
| Amsterdam Cognition Scan | x |  |
| Faeces sample * | x |  |
| Device-measured physical activity (Fitbit activity tracker) | x |  |
| Self-reported sick leave | x | x |

* Stockholm sites only

## Data management

All data are registered using an electronic Case Report Form (eCRF). Monitoring is performed according to Good Clinical Practice (GCP) guidelines. The eCRF provides data on age, demographics, background medical data, and initial tumour and lymph node characteristics deriving from clinical, radiological and histopathological assessment, details concerning type, dose and duration of neoadjuvant and adjuvant systemic therapy, as well as histopathological results at surgery and data on follow-up. Data are managed by the Clinical Trial Office at Centre for Clinical Cancer Studies, Karolinska University Hospital, Stockholm, Sweden. Security is comparable to bank security with encrypted data.

Recorded information is pseudonymised and the key kept at each responsible site. Keys may however need to be transferred to trial staff for quality assurance purposes such as central review of pathology slides and central review of imaging. Information is confidential and the database is privacy-protected; i.e., no data can be traced back to the patient in research reports and no unauthorized individuals may have access to the data about individuals in the database. The database will be maintained until further notice (at least 20 years after inclusion of the last patient) and be reported in accordance with General Data Protection Regulation (GDPR). The authority responsible for the database is Karolinska Institutet, Stockholm, Sweden.

The Neo-ACT trial is registered at www.clinicaltrials.gov (NCT05184582).

## Monitoring and follow-up

This prospective trial is conducted according to GCP guidelines and monitored by the Clinical Trial Office at Centre for Clinical Cancer Studies, Karolinska University Hospital, Stockholm, Sweden, for Swedish sites, and further CROs in further countries participating in this trial. CROs will be monitoring inclusion and exclusion criteria as well as completeness and accuracy of data recorded in the eCRF by regular on-site visits. To this end, participating units will grant access to patient medical files in due time on request. Patients are informed about monitoring procedures and medical file access in the patient information leaflet and grant their consent to these by signing the consent form.

Each patient is followed up for two years regarding secondary endpoints. Follow-up can be conducted as telephone call or by post, and also includes access to the participant’s medical file in order to check for survival and recurrence. Follow-up must be performed within +/- two months from the surgery date, and data are to be completed in the eCRF within one month from the follow-up date. Long-term follow-up regarding survival and recurrence is conducted via national registers.

Participating sites that do not adhere to GCP guidelines or to the agreements stated in the contract signed between the medical responsible at the individual site and the Clinical Trial Office may be excluded from this trial.

## End of trial

The trial will end for each participant followed for two years after the date of surgery, but also for participants who die, withdraw consent or are lost to follow-up.

## Adverse Events

An Adverse Event (AE) is any untoward medical occurrence in a patient or a clinical investigation subject which does not necessarily have a causal relationship with the trial intervention. An AE can therefore be any unfavourable or unintended finding, symptom, or disease temporally associated with a trial intervention, whether or not related to it.

Patients will be instructed to report the occurrence of AEs in the mobile application. The local investigator will only document AEs of specific interest in relation to the intervention, which are exercise-related AEs

- requiring treatment or talking to a doctor or other health professional
- causing any persistent worsening of participants’ health or well-being, new occurrence of substantial pain or swelling e.g. muscle tear
- occurring during or after the exercise session and requiring restrictions/alterations or early termination of the exercise, or any treatment or clarification by a physician

As some examples, patient-experienced severe pain after exercise is reported as an AE if it requires contact with a treating physician; muscle tears occurring during the exercise session is reported as AE; dizziness or nausea during the exercise session resulting in early termination of the exercise session is reported as an AE. Changes in the exercise program due to recent oncological treatment or due to presence of metastases are *not* reported as AE. The local investigator assesses and records the AEs observed during the AE reporting period: from the date of patient consent signature up to 24 months after randomization. **No AEs related to NACT should be reported.**

A Serious Adverse Event (SAE) (ClinO, Art. 63) is any untoward medical occurrence that results in death or is life-threatening, requires in-patient hospitalisation or prolongation of existing hospitalisation, results in persistent or significant disability or incapacity, or causes a congenital anomaly or birth defect. Local investigators make a causality assessment of the event to the trial intervention (see table below). Any event assessed as possibly, probably or definitely related is classified as related to the trial intervention. **No AEs related to NACT or adjuvant therapy should be documented as an SAE.**

Causality of AEs to trial intervention is assessed according to the following scale:

| Not related | The AE is clearly not related to the trial intervention. It is independent of trial intervention, or evidence prevails that it is related to other aetiology. |
| --- | --- |
| Unlikely | The AE is doubtfully related to the trial intervention. Temporal association between the AE and the trial intervention and the nature of the AE is such that the trial intervention is not likely to have had any reasonable association with the observed AE (cause and effect relationship improbable but not impossible). |
| Possibly | The AE may be related to the trial intervention. Less clear temporal association; other aetiologies also possible. |
| Probably | The AE is likely related to the trial intervention. Clear-cut temporal association; a potential alternative aetiology is not apparent. |
| Definitely | The ae is clearly related to the trial intervention. Clear-cut temporal association, and no other possible cause. |

Severity assessment: Local investigators make a severity assessment of the event according to the Common Terminology Criteria for Adverse Events Version 5 published November 27, 2017^34^.

| Grade 1 | Mild; asymptomatic or mild symptoms; clinical or diagnostic observations only;  intervention not indicated. |
| --- | --- |
| Grade 2 | Moderate; minimal, local or non-invasive intervention indicated; limiting age-appropriate instrumental ADL. |
| Grade 3 | Severe or medically significant but not immediately life-threatening;  hospitalization or prolongation of hospitalization indicated; disabling; limiting  self-care ADL. |
| Grade 4 | Life-threatening consequences; urgent intervention indicated. |
| Grade 5 | Death related to AE. |

A Semi-colon indicates ‘or’ within the description of the grade. ADL: activities of daily living

AEs have to be reported as SAE only when they are related (possibly, probably, definitely) to trial intervention. AEs related to NACT or adjuvant therapy are not considered as a SAE and are therefore exempted from expedited reporting. Trial intervention-related SAEs are documented and reported immediately (within a maximum of 24 hours) to the Principal Investigators.

All AEs will be followed until they have abated, or until a stable situation has been reached. Depending on the event, follow-up may require additional tests or medical procedures as indicated, and/or referral to a general physician or a medical specialist.

## Estimated sample size and power

Patients will be randomized in a 1:1 fashion. It is anticipated that the rate of the primary endpoint pCR will be approximately 30% in the control arm. We aim to increase the rate of pCR in the experimental arm to 40%, i.e. a 10 percentage points increase, which is regarded clinically relevant since it would translate into improved disease-related outcomes. With a power of 80% and an alpha of 5%, a total of 712 patients have to be included; 356 in each arm. Accounting for a drop-out of 10%, we aim to include 790 patients. Stratification at the moment of computerized randomization will be done based on site of treatment (hospital) and biological tumour subtype (ER+HER2-, ER+HER2+, ER-HER2+, ER-HER2-).

## Statistical analysis plan

All outcomes will primarily be analysed using an intention-to-treat approach, i.e. all study subjects will belong to the treatment group (exercise intervention or control) they were assigned to, disregarding compliance. As sensitivity analysis, all outcomes will also be analysed using a per-protocol approach, meaning that participants in the intervention group who comply with less than 65% of the prescribed physical exercise program or complete less than 40% of the planned neo-adjuvant systemic therapy (around two 3-weekly courses) will be excluded from analysis.

*Covariate adjustment in statistical models*

Guidelines from the European Medicines Agency (EMA) regarding statistics and covariate adjustments recommend that factors that are used to stratify the randomization, i.e. site of treatment and biological tumour type in this trial, should be accounted for in the statistical analysis^35,36^. However, it is also recognized that “limited numbers of subjects per centre will make it impracticable to include the centre effects in the statistical model”. In this trial it is very likely that some of the centres will contribute few patients, which may cause problems in some of the statistical models, in particular for the categorical outcomes (such as the primary endpoint, pCR) where the variability in the outcome may be too small within each site. Hence, the statistical models, described below, will primarily be adjusted for biological tumour type and not for study site. If appropriate and if the data allows (i.e. no convergence problems and not too small subgroups), the site effect will also be incorporated in the models as a random effect in the mixed models and as a fixed factor in the remaining logistic regression models.

#### Primary endpoint (pCR)

The primary outcome in this trial (pCR) is dichotomous, and will be assessed through histopathological examination of the surgical specimen after completed NACT. Hence, a multivariable logistic regression model, adjusting for biological tumour type (stratification factor in the randomization) will be used. The treatment effect will be assessed in terms of the resulting odds ratio between the two treatment arms together with a 95% confidence interval and a complementary Wald test. A two-sided statistical test with 5% significance level will be used. Furthermore, differences in pCR rates between the two treatment arms will be explored for each biological tumour type subgroup (ER+HER2-, ER+HER2+, ER-HER2+, ER-HER2-), using an interaction between treatment and biological tumour type.

#### Secondary endpoints

1. *Residual Cancer Burden (RCB)*

Residual Cancer Burden (RCB) is measured as four ordered categories (RCB-0 which corresponds to pCR, RCB-I, RCB-II, and RC-III) at histopathological assessment of the surgical specimen. An ordinal regression model, adjusting for biological tumour type (stratification factor in the randomization), will be used to analyse differences between the treatment arms. If the proportional odds assumption of the model is violated, a nominal regression model or a generalized logistic model will be used instead if appropriate.

1. *Objective tumour response*

Response Evaluation Criteria in Solid Tumours (RECIST) will be applied in order to assess change in radiological tumour size (%) from baseline to pre-surgery imaging. RECIST criteria classify response into four ordinal outcome categories: complete response, partial response, stable disease and progressive disease (see page 18)^31^. An ordinal regression model, adjusting for biological tumour type (stratification factor in the randomization), will be used to analyse differences between the treatment arms. If the proportional odds assumption of the model is violated, a nominal regression model or a generalized logistic model will be used instead if appropriate. Tumour size at histopathological assessment of the surgical specimen may be included in comparative analyses. Mean difference in reduction of tumour size between treatment arms will be analysed with an ANOVA, including biological subtype as a factor in the model.

1. *Survival outcomes*

All-cause, breast cancer-specific and recurrence-free survival will be analysed at 2, 5 and 10 years. Contralateral breast cancer is not regarded an event. Difference in time-to-event outcomes between treatment arms will be compared. The mortality outcomes are defined as time from date of randomisation to death (from any cause or breast cancer as underlying cause, respectively) or censoring at end of follow-up. For recurrence-free survival, the outcome is measured as time from date of randomisation to breast cancer relapse or censoring at end of follow-up. Survival estimates after 2, 5 and 10 years of follow-up will be compared using Kaplan-Meier estimates. Furthermore, a Cox regression model, adjusting for biological subtype will also be used.

1. *Health-related quality of life*

All health-related quality of life outcomes are measured as scores that range from 0 to 100, hence the outcomes are continuous. Both the global QoL score as well as the 17 subscales (8 scales reflecting symptoms and 9 reflecting function) will be assessed at four time points: baseline, pre-surgery, 1 and 2 years after surgery. To evaluate QoL scores at each time point both within and between the treatment groups, a mixed model for repeated measures (MMRM) will be used. Treatment, visit, treatment visit interaction and stratification factor biological tumour type will be included in the model as fixed effects, and patient as a random effect. An unstructured covariance matrix will be used to model the within-subject error. If the fit of the chosen structure fails to converge, the following covariance structures will be evaluated, in order, until convergence is reached: toeplitz with heterogeneity, autoregressive with heterogeneity, toeplitz, autoregressive and compound symmetry. Furthermore, differences in change from baseline between the treatment arms will be assessed at each time point by constructing relevant contrasts of the estimated regression coefficients.

1. *Self-reported physical activity*

Physical activity is reported using the Godin-Shephard Leisure-Time Physical Activity Questionnaire (GSLTPAQ) as total minutes per week of training in three intensity levels (light, moderate and intensive) and for weight training. Hence, the outcome is continuous. A mixed model for repeated measures (MMRM) will be used to assess physical activity at four different time points: baseline, pre-surgery, 1 and 2 years after surgery). The model will include treatment, visit, treatment-visit interaction and the stratification factor biological tumour type as fixed effects, and patient as a random effect. An unstructured covariance matrix will be used to model the within-subject error. If the fit of the chosen structure fails to converge, the following covariance structures will be evaluated, in order, until convergence is reached: toeplitz with heterogeneity, autoregressive with heterogeneity, toeplitz, autoregressive and compound symmetry. Physical exercise will be assessed both within and between the treatment groups. Furthermore, differences in change from baseline between the treatment arms will be evaluated at each time point by constructing relevant contrasts of the estimated regression coefficients.

An additional analysis, using a dichotomous outcome that classifies patients as *active* and *insufficiently active* based on a score generated from GSLTPAQ, will be performed. Here, a generalized linear mixed model (GLMM) for repeated measures with a logit link function will be implemented. Activity will be assessed at baseline, pre-surgery and at 1- and 2-year follow-up, both within and between treatment arms. An unstructured covariance matrix will be used to model the within-subject error. If the fit of the chosen structure fails to converge, the following covariance structures will be evaluated, in order, until convergence is reached: toeplitz with heterogeneity, autoregressive with heterogeneity, toeplitz, autoregressive and compound symmetry.

1. *Toxicity-related outcomes*

The following toxicity-related outcomes will be assessed: a, chemotherapy completion rates, b, number of unplanned hospital admissions, c, cognitive function (measured at baseline and one year after surgery), d, cardiotoxicity and e, sick leave.

1. Chemotherapy completion rates, i.e. the proportion of participants receiving the planned number of treatments including potential adjuvant chemotherapy, is a dichotomous outcome (yes/no). Both neoadjuvant and adjuvant treatment will be assessed separately (regarded as two independent measurements) in multivariable logistic regression models, adjusting for biological tumour type (stratification factor in the randomization). The treatment effect will be assessed in terms of a resulting odds ratio and a complementary Wald test.
2. For the count outcome number of unplanned hospital admissions, we will use a Poisson regression model, adjusting biological tumour type. If overdispersion is present, a negative binomial regression will be considered instead. Results from the final model (Poisson or negative binomial, whichever fits the data best) will be presented as incidence ratios (IR) together with confidence intervals and a Wald test of the treatment effect.
3. Objective cognitive dysfunction is measured by an online neuropsychological test (Amsterdam Cognition Scan) at baseline and 1-year follow-up. The total Amsterdam Cognition Scan (ACS) score, calculated as the mean of the (reversed) z-scores of all main online neuropsychological outcome measures, will be of primary interest. Furthermore, separate z-scores of the cognitive tests will also be assessed. Hence, the measured cognitive dysfunction outcomes, measured at baseline and at 1-year follow-up, are continuous variables. A mixed model for repeated measures (MMRM) will be used to assess change from baseline of ACS scores, which is the primary interest. The model will include treatment, visit and the stratification factor biological tumour type as fixed effects, and patient as a random effect. An unstructured covariance matrix will be used to model the within-subject error. If the fit of the chosen structure fails to converge, the following covariance structures will be evaluated, in order, until convergence is reached: toeplitz with heterogeneity, autoregressive with heterogeneity, toeplitz, autoregressive and compound symmetry. ACS scores will be assessed both within and between the treatment groups.
4. Cardiovascular toxicity is a dichotomous outcome (yes/no) that will be evaluated only in the subgroup of patients with HER2-positive tumours who are treated with anti-HER2 therapies (approximately 30% of the study population). A multivariable logistic regression model, adjusting for biological tumour type will be used to assess differences between the treatment arms.
5. Sick leave is a patient-reported singular measurement at pre-surgery and at 1- and 2-year follow-up (% sick leave). Mean sick leave proportions will be compared between the treatment arms at each time point using a mixed model for repeated measures (MMRM). Treatment, visit, treatment visit interaction and the stratification factor biological tumour type will be included in the model as fixed effects, and patient as a random effect. An unstructured covariance matrix will be used to model the within-subject error. If the fit of the chosen structure fails to converge, the following covariance structures will be evaluated, in order, until convergence is reached: toeplitz with heterogeneity, autoregressive with heterogeneity, toeplitz, autoregressive and compound symmetry. Additionally, since the outcome is most likely not normally distributed, a non-parametric Kruskal-Walllis test will be performed at each time as a sensitivity analysis.
6. *Device-measured physical activity level*

Several continuous outcome measures from the Fitbit activity tracker will be assessed: 1) METs (metabolic equivalents) in low, medium and high intensity activities, respectively, 2) step counts per day and 3) daily active minutes. The main interest is to see if there is a difference between the treatment arms regarding change of physical activity from baseline to pre-surgery (i.e. after intervention and chemotherapy), but also at 1-year follow-up. A mixed model for repeated measures (MMRM) with treatment, visit and the stratification factor biological subtype as fixed effects, and patient as a random effect. An unstructured covariance matrix will be used to model the within-subject error. If the fit of the chosen structure fails to converge, the following covariance structures will be tried, in order, until convergence is reached: toeplitz with heterogeneity, autoregressive with heterogeneity, toeplitz, autoregressive and compound symmetry. Additionally, heart rates at equivalent exertion levels (assessed on the Borg scale) during physical activity will be explored.

1. *Muscle strength*

The muscle strength is measured on a continuous score on each arm and leg. The main interest is to see if there is a change in strength after intervention, i.e. change from baseline to pre-surgery. A mixed model for repeated measures (MMRM) with treatment, visit and the stratification factor biological tumour type as fixed effects, and patient as a random effect. An unstructured covariance matrix will be used to model the within-subject error. If the fit of the chosen structure fails to converge, the following covariance structures will be tried, in order, until convergence is reached: toeplitz with heterogeneity, autoregressive with heterogeneity, toeplitz, autoregressive and compound symmetry.

1. *Cardiorespiratory fitness*

The Åstrand submaximal cycle test renders a continuous outcome. The main interest is to see if there is a change in cardiorespiratory fitness after intervention, i.e. change from baseline to pre-surgery. A mixed model for repeated measures (MMRM) with treatment, visit and the stratification factor biological tumour type as fixed effects, and patient as a random effect. An unstructured covariance matrix will be used to model the within-subject error. If the fit of the chosen structure fails to converge, the following covariance structures will be tried, in order, until convergence is reached: toeplitz with heterogeneity, autoregressive with heterogeneity, toeplitz, autoregressive and compound symmetry.

#### Reporting

For the secondary outcomes, the differences between the treatment arms will be reported as an effect measure (e.g. odds ratio or mean difference) together with a complementary confidence interval and a two-sided statistical test of the effect parameter. A Bonferroni corrected significance level will be used.

#### Handling missing values and deaths

Experience from previous studies have shown that this group of patients are in general highly motivated to fully participate in trials, including answering questionnaires, undergo physical exams and other assessments. Hence, it is anticipated that the dropout rate and the number of missing values will be low in both treatment arms and that this will be a minor problem in this study. Furthermore, the number of deaths during this relatively short follow-up period is also expected to be low. Nevertheless, sensitivity analysis will be performed where missing values will be assumed to be missing at random, MAR. This assumption will be already accommodated in the mixed models where repeated measurement outcomes will be analysed. For the remaining outcomes multiple imputations, assuming MAR will be performed.

A data monitoring committee consisting of three independent experts will perform a safety analysis with the purpose to assess the recruitment to the trial and the rate of adverse events, and to make sure that patients in the intervention group do not appear to fare significantly worse than patients in the standard of care group. The committee may recommend terminating the study if a significant benefit in favour of one group is shown, such that the HR for intervention versus standard of care significantly (p=0.001) exceeds 1, if the recruitment is so low that that the necessary number of events is unlikely to be reached or if there are serious concerns about unexpected AEs in the intervention group. If the committee determines that it is safe to proceed with the study, the results of the analysis will remain unknown to everyone except the committee members.

## Ethical considerations

The original version of this trial protocol has been approved by the Ethics Committee of the Karolinska Institutet, Stockholm (Dnr 2022-02084-01).

Physical exercise during chemotherapy is deemed feasible and safe^19^. Potential adverse events, such as cardiovascular and musculoskeletal symptoms, will be recorded throughout the NACT period and participants who experience serious adverse events will discontinue trial participation. Adverse events that are ascribed NACT but not the trial intervention will be handled in accordance to clinical routine by the responsible oncologists. There is no evidence that the intervention may lead to inferior results concerning the primary or secondary endpoints. Instead, there are data from studies in the adjuvant setting showing a preserved health-related quality of life, lower rate of self-reported symptoms related to treatment, and higher chemotherapy completion rates. In addition, this trial offers patient empowerment that is generally experienced as a positive aspect of treatment. Therefore, participation in this trial is judged ethically highly feasible.

# Withdrawal

Patients who wish to withdraw from the trial may do so at any time, without providing a reason. Data already included in previous eCRFs will be included in the analysis if the participant does not explicitly wish to have his/her data excluded from analysis. Ceasing participation will be recorded in the eCRF.

# Publication policy

Before the collaborative publication of the main outcome from the entire cohort, no other publication regarding the primary outcome on the whole or parts of the cohort can be attempted. Publications of secondary endpoints or the trial protocol may be undertaken prior to the main publication. Each principal investigator is a co-author in any publication reporting on pre-planned analyses from the Neo-ACT trial, that is, any report on primary or secondary outcomes, protocol, safety reports or translational substudies. Any further analysis using data from the Neo-ACT trial must first get the permission from the principal investigators. The principal investigators must in such case be permitted to take a more active part and thus fulfil the ICMJE criteria for authorship. Any investigators not fulfilling ICMJE criteria for authorship must be individually named in Acknowledgements.

# Time plan

Based on our experience in the OptiTrain trial, the rate of informed consent in patients up to the age of 70 years is about 50%. Participation rates may potentially rise in this trial since awareness of the benefits of physical exercise in the context of cancer has increased and since Neo-ACT for the first time offers a real-time measurement of the anti-tumoral effects of physical exercise which may offer the participants prognostic gains. However, competing oncological trials investigating pCR rates may negatively affect participation rates: Currently, in spring of 2022, the Swedish PREDIX Luminal B trial is closed, while the ongoing Nordic Trip trial includes patients with T2-3 or node-positive triple-negative breast cancer. Further potential Swedish trials, such as the ARIADNE trial in HER2+ patients, may be initiated in the near future.

In 2020, the proposed trial sites (at the time of writing 10 Swedish and 1-7 Finnish sites) registered over 900 newly diagnosed BC patients receiving NACT. The proportion of NACT has been increasing over the last years, and a decline is not anticipated. With an estimated participation rate of one third, more than 300 patients may be enrolled per year. Accrual rates will be monitored each month and further sites may be opened if accrual drops below estimated rates. The Neo-ACT trial is planned to open for recruitment in September 2022, and it is estimated that inclusion may be completed in December 2025. An independent interim safety analysis is performed after recruitment of 200 patients or after two years, whatever occurs first. The primary endpoint is analysed once all participants have had surgery. Follow-up will be two years per individual.

# Translational research

The following translational projects are proposed. This list does not claim to be complete, and further translational projects may be added to the trial at any time point.

## Faeces microbiome

A growing research interest has arisen in the association between the composition of the intestinal microbiome and development and treatment-related outcomes of cancer. Especially the association between response to immune checkpoint inhibitors (ICI) and the intestinal microbiome is of great relevance, where pre-clinical and clinical studies have shown that presence of certain forms of microbiome components as well as recent and current use of antibiotics are associated with a lack of ICI treatment effect^37,38^. Intervention studies using faeces modification with the aim to change the intestinal microbiome and thereby potentially improve the response to ICI treatment are ongoing. First results are promising^39^.

Physical exercise increases the number of beneficial microbial species, leads to an enrichment of the microflora diversity, and an improved development of commensal bacteria^40^. Proposed mechanisms include the release of neuroendocrine and immune-modulatory factors, which in turn may lower inflammatory and oxidative stress, and thereby beneficially affect metabolic disorders^41^.

The interplay between physical exercise, cancer and the intestinal microbiome is insufficiently explored. In a study in 15 patients who underwent resection for early-stage lung cancer, the gut-lung axis was investigated through paired faeces samples pre- and post-surgery. Changes in microbial community functional profiles were observed between both time points, as well as an association between functional capacity (VO2) and an increase in certain microbiotic species^42^. A randomised controlled trial was recently initiated exploring the impact of a three-month exercise programme for men with high-risk prostate cancer on androgen deprivation therapy on the intestinal microbiome and gut health. Gut health and gut function assessed via faecal samples is the primary endpoint whereas secondary endpoints include self-reported quality of life^43^.

In Neo-ACT, clinical data on antibiotic and proton-pump inhibitor (self-reported) use up to six months pre-baseline will be registered. Faeces will be collected at baseline, before surgery and at one-year follow-up. Changes in microbiome will be compared between the randomization groups and over time.

## Intratumoral and circulating Natural Killer (NK) cells

Transplantation studies show that NK cells are involved in tumor rejection and protection from relapse, supporting the therapeutic potential of NK cells in tumor eradication ^44,45^. Despite these encouraging findings, NK cell therapies are limited by the lack of antigen specificity. Also, similar to T cells, resistance to NK cell-mediated killing may develop due to the recruitment and differentiation of immune suppressive cells, including regulatory T cells (Treg) and myeloid derived suppressor cells (MDSC), and overexpression of immune inhibitory checkpoint proteins in the tumor microenvironment (TME). The adaptive NK (aNK) cell subset, defined by the expression of the maturation marker CD57, the activation receptor NKG2C, and the downregulation of several signaling molecules including PLZF, Syk, and FCεR1γ, is able to resist the TME suppression in hematological malignancies. Mechanisms that spare aNK cells from immune suppression by MDSC and Treg involves low expression of the checkpoint molecules T cell immunoglobulin and ITIM domain (TIGIT), programed death receptor (PD-1), NKG2A, and the IL-1R8 ^46,47^.

Samples from tumor and blood will be collected at two time points; at baseline prior to start of chemotherapy as well as at surgery. For this purpose, one fresh core biopsy and two tubes of heparin blood are utilized.

**The aim is to** identify biomarkers/predictive markers associated with aNK cells in patients with breast cancer undergoing NACT with and without physical exercise. Examinations will enable identification of new biomarkers that determine the endpoint of NACT and long-term clinical responses. **The specific aims are to c**haracterize aNK cells in breast cancer and exploit whether NACT and physical exercise harness aNK cell memory and to investigate aNK cell interaction with other cells in the TME comparing the two trial groups.

## Tumour gene profiling

RNA and DNA will be extracted from RNAlater and/or FFPE-preserved biopsies from the primary tumour (preoperative core biopsies as well as surgically resected tumours after NACT), axillary metastases and distant/local recurrences for further analysis. Gene expression profiling will be performed to identify intrinsic subtypes, molecular signalling pathways and additional programs or genes associated with pathological response and with additional biomarkers and physical activity. DNA sequencing (exome, whole genome or targeted panels) will investigate any genomic variants.

## Tumour microenvironment

Baseline presence and composition of tumour-infiltrating lymphocytes (TILs) are prognostic in triple-negative and HER2-positive breast cancers treated with neoadjuvant systemic therapy, and predictive for pCR^48,49^. TILs will be assessed by pathologists in the untreated primary tumour biopsy and the surgical specimen according to international guidelines (<https://www.tilsinbreastcancer.org/>). Even beyond TILs, the tumor microenvironment plays a crucial role in the response to neoadjuvant chemotherapy. Our hypothesis is that the study intervention (physical exercise) can improve pCR rates in tumors that are immunologically cold at baseline.

A study-specific tumor biopsy will be obtained from all study participants before start of neoadjuvant therapy (baseline biopsy) and will be formalin-fixed and paraffin embedded (FFPE). Similarly, tumor material will be obtained from the surgical specimen. Whole sections will be used for H&E staining and subsequently scanning for digital pathology and deep-learning image analyses. Tumor infiltrating lymphocytes (TILs) will be correlated with clinical characteristics and outcomes. Tissue microarrays (TMAs) will be fabricated and will be used for multiplex immunofluorescence assays for characterization of tumor cells, immune and other cell types in the tumor stroma both at baseline and at surgery. For this purpose. the 7-color IHC method (Opal 7 Solid Tumor Immunology Kit, PerkinElmer) using a panel of lymphocytic and macrophage markers and an additional panel of fibroblast, blood vessel and mesenchymal cell markers will be utilized. Multispectral fluorescent scanning (Vectra 3 Quantitative Pathology Systems) and image analysis with compatible software will be used for the visualization and quantitation of the multiple markers in a spatial tissue context.

## Circulating tumour cells and tumour DNA

DNA analysis will be performed in blood plasma specimens from the patients (liquid biopsy). Genomic variants will be compared in different localisations and sample types to investigate clonality and tumour evolution.

## The effects of human adipocytes on breast cancer progression and metastasis

The worldwide prevalence of obesity nearly tripled during the past decades. Overall, in 2016 about 13% of the world’s adult population were obese. Obesity rates in Sweden have also increased, with almost 20% of Swedes between 45-84 years old being obese. The global obesity rate in women is projected to reach 21% by 2025 and this is particularly alarming considering that 55% of all female cancers have an obesity-associated mechanism^50^. Obesity is defined as an abnormal excessive fat accumulation that causes a health risk. Body Mass Index (BMI) is a useful index of weight-for-height that is commonly used to classify this pathology. Obesity increases the risk for many types of cancer and is associated with poor outcomes. Despite a strong association with obesity, most current cancer treatments do not take into consideration the ongoing obesity epidemic. Whilst preventative measures, such as promoting weight loss should be conducted, often once cancer has been detected there may not be time to lose weight before treatment. As such, there is a need to develop specific drug targets that could be leveraged to address the obesity component of the disease.

In breast cancer obesity is only associated with an increased incidence of post-menopausal breast cancer, whilst obesity is a risk factor for progression in all breast cancer subtypes^51^. An important and negative predictor of cancer survival is metastasis. The mechanism underlying the metastatic spread of cancer including Epithelial-Mesenchymal-Transition (EMT), cell migration, progression, and dissemination remains unclear. The EMT is a critical tumour cell plasticity and dedifferentiation program, by which epithelial cells acquire pro-migratory and invasive mesenchymal properties. The effects of non-cancer associated adipocytes on promoting EMT and cancer progression in lean and obese individuals remain largely unknown^52^.

In the Neo-ACT trial, we will acquire fresh fat tissues from the operated breast regardless of the NACT response of the individual patient. We will address the following questions as specific study aims:

1. Determine the potential effect of NACT on breast tissue fat cells senescence.

2. Determine whether adipocyte senescence level correlates with exercise and leads to different secretion patterns of various soluble factors and therefore enhances the attraction/infiltration of immune cells.

3. Determine whether factors secreted by adipocytes in resting versus exercising individuals are correlated with treatment response.

4. Determine whether patients from different BMI groups (both before and after exercises) have distinct response patterns and if adipose tissues play a role in it.

5. Identify possible biomarkers for breast cancer-associated adipocytes and their possible implications in breast cancer treatment regimen design (eg. CDK4/6 inhibitors).

The methods include basic cell and molecular experiment procedures including but not limited to culturing of patient adipocytes, staining, as well as RNAseq analysis. The results can be analysed together with the RNAseq results of biopsy/residual tumours, as well as the flow cytometry data to find connections with immune infiltration patterns. Blood testing results will be vital for subgroup patient populations and correlation analysis.

# References

1. RCC. *Nationellt vårdprogram [Swedish National Guidelines].* Regionala cancercentrum i samverkan;2020.

2. Burstein HJ, Curigliano G, Thurlimann B, et al. Customizing local and systemic therapies for women with early breast cancer: the St. Gallen International Consensus Guidelines for treatment of early breast cancer 2021. *Ann Oncol.* 2021;32(10):1216-1235.

3. Cortazar P, Zhang L, Untch M, et al. Pathological complete response and long-term clinical benefit in breast cancer: the CTNeoBC pooled analysis. *Lancet.* 2014;384(9938):164-172.

4. Gianni L, Pienkowski T, Im YH, et al. Efficacy and safety of neoadjuvant pertuzumab and trastuzumab in women with locally advanced, inflammatory, or early HER2-positive breast cancer (NeoSphere): a randomised multicentre, open-label, phase 2 trial. *Lancet Oncol.* 2012;13(1):25-32.

5. Early Breast Cancer Trialists' Collaborative G. Long-term outcomes for neoadjuvant versus adjuvant chemotherapy in early breast cancer: meta-analysis of individual patient data from ten randomised trials. *Lancet Oncol.* 2018;19(1):27-39.

6. Schmid P, Cortes J, Pusztai L, et al. Pembrolizumab for Early Triple-Negative Breast Cancer. *N Engl J Med.* 2020;382(9):810-821.

7. Masuda N, Lee SJ, Ohtani S, et al. Adjuvant Capecitabine for Breast Cancer after Preoperative Chemotherapy. *N Engl J Med.* 2017;376(22):2147-2159.

8. von Minckwitz G, Huang CS, Mano MS, et al. Trastuzumab Emtansine for Residual Invasive HER2-Positive Breast Cancer. *N Engl J Med.* 2019;380(7):617-628.

9. Pan SY, DesMeules M. Energy intake, physical activity, energy balance, and cancer: epidemiologic evidence. *Methods Mol Biol.* 2009;472:191-215.

10. King MC, Marks JH, Mandell JB, New York Breast Cancer Study G. Breast and ovarian cancer risks due to inherited mutations in BRCA1 and BRCA2. *Science.* 2003;302(5645):643-646.

11. Betof AS, Dewhirst MW, Jones LW. Effects and potential mechanisms of exercise training on cancer progression: a translational perspective. *Brain Behav Immun.* 2013;30 Suppl:S75-87.

12. Schadler KL, Thomas NJ, Galie PA, et al. Tumor vessel normalization after aerobic exercise enhances chemotherapeutic efficacy. *Oncotarget.* 2016;7(40):65429-65440.

13. McCullough DJ, Stabley JN, Siemann DW, Behnke BJ. Modulation of blood flow, hypoxia, and vascular function in orthotopic prostate tumors during exercise. *J Natl Cancer Inst.* 2014;106(4):dju036.

14. Rogers CJ, Zaharoff DA, Hance KW, et al. Exercise enhances vaccine-induced antigen-specific T cell responses. *Vaccine.* 2008;26(42):5407-5415.

15. Savas P, Salgado R, Denkert C, et al. Clinical relevance of host immunity in breast cancer: from TILs to the clinic. *Nat Rev Clin Oncol.* 2016;13(4):228-241.

16. Gustafson MP, DiCostanzo AC, Wheatley CM, et al. A systems biology approach to investigating the influence of exercise and fitness on the composition of leukocytes in peripheral blood. *J Immunother Cancer.* 2017;5:30.

17. Hojman P, Gehl J, Christensen JF, Pedersen BK. Molecular Mechanisms Linking Exercise to Cancer Prevention and Treatment. *Cell Metab.* 2018;27(1):10-21.

18. Pedersen L, Idorn M, Olofsson GH, et al. Voluntary Running Suppresses Tumor Growth through Epinephrine- and IL-6-Dependent NK Cell Mobilization and Redistribution. *Cell Metab.* 2016;23(3):554-562.

19. Zylstra J, Whyte GP, Beckmann K, et al. Exercise prehabilitation during neoadjuvant chemotherapy may enhance tumour regression in oesophageal cancer: results from a prospective non-randomised trial. *Br J Sports Med.* 2022;56(7):402-409.

20. Loughney L, West MA, Kemp GJ, Grocott MP, Jack S. Exercise intervention in people with cancer undergoing neoadjuvant cancer treatment and surgery: A systematic review. *Eur J Surg Oncol.* 2016;42(1):28-38.

21. Sturgeon KM, Smith AM, Federici EH, et al. Feasibility of a tailored home-based exercise intervention during neoadjuvant chemotherapy in breast cancer patients. *BMC Sports Sci Med Rehabil.* 2022;14(1):31.

22. Cormie P, Zopf EM, Zhang X, Schmitz KH. The Impact of Exercise on Cancer Mortality, Recurrence, and Treatment-Related Adverse Effects. *Epidemiol Rev.* 2017;39(1):71-92.

23. Courneya KS, Segal RJ, McKenzie DC, et al. Effects of exercise during adjuvant chemotherapy on breast cancer outcomes. *Med Sci Sports Exerc.* 2014;46(9):1744-1751.

24. Hayes SC, Steele ML, Spence RR, et al. Exercise following breast cancer: exploratory survival analyses of two randomised, controlled trials. *Breast Cancer Res Treat.* 2018;167(2):505-514.

25. Mijwel S, Backman M, Bolam KA, et al. Highly favorable physiological responses to concurrent resistance and high-intensity interval training during chemotherapy: the OptiTrain breast cancer trial. *Breast Cancer Res Treat.* 2018;169(1):93-103.

26. Mijwel S, Cardinale DA, Norrbom J, et al. Exercise training during chemotherapy preserves skeletal muscle fiber area, capillarization, and mitochondrial content in patients with breast cancer. *FASEB J.* 2018;32(10):5495-5505.

27. Anampa J, Makower D, Sparano JA. Progress in adjuvant chemotherapy for breast cancer: an overview. *BMC Med.* 2015;13:195.

28. van Waart H, Stuiver MM, van Harten WH, et al. Effect of Low-Intensity Physical Activity and Moderate- to High-Intensity Physical Exercise During Adjuvant Chemotherapy on Physical Fitness, Fatigue, and Chemotherapy Completion Rates: Results of the PACES Randomized Clinical Trial. *J Clin Oncol.* 2015;33(17):1918-1927.

29. Xu L, Li F, Zhou C, Li J, Hong C, Tong Q. The effect of mobile applications for improving adherence in cardiac rehabilitation: a systematic review and meta-analysis. *BMC Cardiovasc Disord.* 2019;19(1):166.

30. Groen WG, van Harten WH, Vallance JK. Systematic review and meta-analysis of distance-based physical activity interventions for cancer survivors (2013-2018): We still haven't found what we're looking for. *Cancer Treat Rev.* 2018;69:188-203.

31. Schwartz LH, Seymour L, Litiere S, et al. RECIST 1.1 - Standardisation and disease-specific adaptations: Perspectives from the RECIST Working Group. *Eur J Cancer.* 2016;62:138-145.

32. Tolaney SM, Garrett-Mayer E, White J, et al. Updated Standardized Definitions for Efficacy End Points (STEEP) in Adjuvant Breast Cancer Clinical Trials: STEEP Version 2.0. *J Clin Oncol.* 2021;39(24):2720-2731.

33. Feenstra HEM, Murre JMJ, Vermeulen IE, Kieffer JM, Schagen SB. Reliability and validity of a self-administered tool for online neuropsychological testing: The Amsterdam Cognition Scan. *J Clin Exp Neuropsychol.* 2018;40(3):253-273.

34. SERVICES UDOHAH. Common Terminology Criteria for Adverse Events (CTCAE) v5.0. In:2017.

35. EMA. Statistical Principles for Clinical Trials. <https://www.ema.europa.eu/en/ich-e9-statistical-principles-clinical-trials#current-version-section>. Published 1998. Accessed.

36. EMA. Guideline on adjustment for baseline covariates in clinical trials. <https://www.ema.europa.eu/en/documents/scientific-guideline/guideline-adjustment-baseline-covariates-clinical-trials_en.pdf>. Published 2015. Accessed.

37. Gopalakrishnan V, Spencer CN, Nezi L, et al. Gut microbiome modulates response to anti-PD-1 immunotherapy in melanoma patients. *Science.* 2018;359(6371):97-103.

38. Zitvogel L, Ayyoub M, Routy B, Kroemer G. Microbiome and Anticancer Immunosurveillance. *Cell.* 2016;165(2):276-287.

39. Routy B, Le Chatelier E, Derosa L, et al. Gut microbiome influences efficacy of PD-1-based immunotherapy against epithelial tumors. *Science.* 2018;359(6371):91-97.

40. Monda V, Villano I, Messina A, et al. Exercise Modifies the Gut Microbiota with Positive Health Effects. *Oxid Med Cell Longev.* 2017;2017:3831972.

41. Sohail MU, Yassine HM, Sohail A, Thani AAA. Impact of Physical Exercise on Gut Microbiome, Inflammation, and the Pathobiology of Metabolic Disorders. *Rev Diabet Stud.* 2019;15:35-48.

42. Marfil-Sanchez A, Seelbinder B, Ni Y, et al. Gut microbiome functionality might be associated with exercise tolerance and recurrence of resected early-stage lung cancer patients. *PLoS One.* 2021;16(11):e0259898.

43. Newton RU, Christophersen CT, Fairman CM, et al. Does exercise impact gut microbiota composition in men receiving androgen deprivation therapy for prostate cancer? A single-blinded, two-armed, randomised controlled trial. *BMJ Open.* 2019;9(4):e024872.

44. Miller JS, Cooley S, Parham P, et al. Missing KIR ligands are associated with less relapse and increased graft-versus-host disease (GVHD) following unrelated donor allogeneic HCT. *Blood.* 2007;109(11):5058-5061.

45. Ruggeri L, Capanni M, Urbani E, et al. Effectiveness of donor natural killer cell alloreactivity in mismatched hematopoietic transplants. *Science.* 2002;295(5562):2097-2100.

46. Luu TT, Ganesan S, Wagner AK, et al. Independent control of natural killer cell responsiveness and homeostasis at steady-state by CD11c+dendritic cells. *Scientific Reports.* 2016;6.

47. Sarhan D, Hippen KL, Lemire A, et al. Adaptive NK Cells Resist Regulatory T-cell Suppression Driven by IL37. *Cancer Immunol Res.* 2018;6(7):766-775.

48. Mao Y, Qu Q, Zhang Y, Liu J, Chen X, Shen K. The value of tumor infiltrating lymphocytes (TILs) for predicting response to neoadjuvant chemotherapy in breast cancer: a systematic review and meta-analysis. *PLoS One.* 2014;9(12):e115103.

49. Hong J, Rui W, Fei X, Chen X, Shen K. Association of tumor-infiltrating lymphocytes before and after neoadjuvant chemotherapy with pathological complete response and prognosis in patients with breast cancer. *Cancer Med.* 2021;10(22):7921-7933.

50. Lengyel E, Makowski L, DiGiovanni J, Kolonin MG. Cancer as a Matter of Fat: The Crosstalk between Adipose Tissue and Tumors. *Trends Cancer.* 2018;4(5):374-384.

51. Renehan AG, Zwahlen M, Egger M. Adiposity and cancer risk: new mechanistic insights from epidemiology. *Nat Rev Cancer.* 2015;15(8):484-498.

52. Dirat B, Bochet L, Dabek M, et al. Cancer-associated adipocytes exhibit an activated phenotype and contribute to breast cancer invasion. *Cancer Res.* 2011;71(7):2455-2465.
